# The application of titanium dioxide, zinc oxide, fullerene, and graphene nanoparticles in photodynamic therapy

**DOI:** 10.1186/s12645-017-0032-2

**Published:** 2017-10-19

**Authors:** Zahraa Youssef, Régis Vanderesse, Ludovic Colombeau, Francis Baros, Thibault Roques-Carmes, Céline Frochot, Habibah Wahab, Joumana Toufaily, Tayssir Hamieh, Samir Acherar, Amirah Mohd Gazzali

**Affiliations:** 1 Laboratoire Réactions et Génie des Procédés, Université de Lorraine-CNRS, UMR 7274, 1 rue Grandville, BP 20451, 54001 Nancy Cedex, France; 2 Laboratoire de Chimie Physique Macromoléculaire, Université de Lorraine-CNRS, UMR 7375, 1 rue Grandville, BP 20451, 54001 Nancy Cedex, France; 3 School of Pharmaceutical Sciences, Universiti Sains Malaysia, 11800 Penang, Malaysia; 4 Laboratory of Materials, Catalysis, Environment and Analytical Methods, Faculty of Sciences I, Lebanese University, Campus Rafic Hariri, Beyrouth, Lebanon

**Keywords:** Nanoparticles, Photodynamic therapy, Titanium dioxide, Zinc oxide, Fullerene, Graphene

## Abstract

Nanoparticles (NPs) have been shown to have good ability to improve the targeting and delivery of therapeutics. In the field of photodynamic therapy (PDT), this targeting advantage of NPs could help ensure drug delivery at specific sites. Among the commonly reported NPs for PDT applications, NPs from zinc oxide, titanium dioxide, and fullerene are commonly reported. In addition, graphene has also been reported to be used as NPs albeit being relatively new to this field. In this context, the present review is organized by these different NPs and contains numerous research works related to PDT applications. The effectiveness of these NPs for PDT is discussed in detail by collecting all essential information described in the literature. The information thus assembled could be useful in designing new NPs specific for PDT and/or PTT applications in the future.

## Background

In current era, the explosion of advancement in nanotechnology has opened up different possibilities of its applications, examples being in drug delivery systems, cosmetics, sunscreens, and electronics. The European Union in 2011 has defined nanomaterials as natural, incidental, or manufactured materials containing particles, in an unbound state, as an aggregate or agglomerate, in which at least 50% of the particles exhibit an external size dimension of between 1 and 100 nm (De Jong and Borm 2008; Shi et al. 2013).

In general, nanomaterials such as nanoparticles (NPs) include polymeric NPs, liposomes (multilayer), lipidic micelles (unilayer), quantum dots and metallic NPs (made from different types of metals such as Au, Fe_2_O_3_, ZnO, TiO_2_…), and graphene (Huang et al. 2012). In addition to these groups of NPs, certain specific structures have also been developed including NPs such as dendrimers, fullerenes, cubosomes, and niosomes (Lohani et al. 2014; Voon et al. 2014). The preparation technique of the NPs differs depending on their structures and need, the most common being nano-precipitation technique, solvent evaporation, and lithography technique, to name a few.

The vast diversified types of NPs available to date provides a possibility to pick and choose the most suitable NPs for specific applications. Indeed, the application of NPs is very wide, ranging from cosmetics, engineering, and medicine through pharmaceuticals, among others. In the interest of our research, NPs are being used in the delivery of photosensitizers (PSs) for photodynamic therapy (PDT), which is a good approach to improving their specific site delivery.

PDT is a relatively new treatment modality that has attracted attention since the past 30 years (Yano et al. 2011). Its principal of treatment necessitates the presence of a PS, light of an appropriate wavelength, and molecular oxygen. Among these three components, PS and light are two modifiable factors, and the development of tumor-specific PS is of interest to many researchers in chemistry and pharmaceutical fields (Olivo et al. 2010).

The modification of PS could be performed by conjugation with targeting moieties or using advanced drug delivery systems such as NPs (Wang et al. 2004; Huang et al. 2012). The PS in this kind of conjugate or system is known as the third-generation PS, which has good potential to improve the targeting and delivery of PS towards the diseased tissues. NPs in particular have shown good ability to enhance the delivery of therapeutics through passive targeting by the enhanced permeability and retention (EPR) effect (Blanco et al. 2015). In PDT, the application of NPs has already been used in the formulation of Visudyne^®^, which is a third-generation PS. In this formulation, its delivery has been shown to be significantly improved (Chang and Yeh 2012).

In the development of third-generation PS, several types of NPs were already being reported in the literature with good potential and positive effect on PDT efficacy. Some representative examples on the use of NPs as anticancer agents are given in the following reviews and can be of interest to the readers (Vanderesse et al. 2011; Couleaud et al. 2011; Benachour et al. 2012; Chouikrat et al. 2012; Monge-Fuentes et al. 2014; Roblero-Bartolon and Ramon-Gallegos 2015; Calixto et al. 2016; Colombeau et al. 2016; Shen et al. 2016; Stallivieri et al. 2016). The nanosize range of NPs is indeed very advantageous because they could penetrate through the fenestration present at the cell junction. Among the different NPs, there are also specific types of particles that have found application in photocatalysis besides being useful in PDT. As examples, the NPs formed by zinc oxide (ZnO), titanium dioxide (TiO_2_), fullerene, and graphene have shown this dual ability based on a huge number of papers published to date. A review by Lucky et al. (2015) has established in detail the different types of biodegradable and non-biodegradable NPs that are currently available. They have also mentioned the works reported on the development of TiO_2_, ZnO, and fullerene NPs as downconverting PSs, but the information gathered was only briefly described.

Besides PDT, hyperthermia (and particularly photothermal therapy, PTT) refers to the use of heat in medicine to increase the temperature of human tissues for therapeutic purposes. For example, it has been used for the treatment of cancer tumors, for more rapidly delivering drugs to cancer tissues by increasing blood flow, or in radiotherapy by sensitizing cancer cells using radiation. Cancer cells are naturally more sensitive to radiation than normal cells. Therefore, several protocols, based on hyperthermia, have been developed to destroy tumor cells irreversibly. Indeed, temperatures ranging from 41 to 47 °C can break the membrane of the cells and denature the proteins. Various laser, microwave, radiofrequency, and ultrasonic methods have been tested to localize and destroy tumors. The main drawbacks are the destruction of healthy tissues close to the tumor and the difficulty in obtaining a uniform temperature in the tumor. In order to achieve better targeting of cancer tissues, some authors have proposed to place photo-absorbing agents in the desired region before irradiation by laser radiation. It has been called photothermal therapy (PTT) because photo-absorbing agents convert light into heat. PTT is a minimally invasive, controllable, and efficient sterilization method. In the presence of an external NIR light source, PTT materials convert light energy into heat energy to kill cells. The most commonly used PTT materials include metal nanoscale materials, such as gold, silver, palladium, copper NPs, graphene and carbon nanotubes, and polymeric NPs.

Dual-modal phototherapeutics that combine PDT and PTT can have synergistic effects that enhance therapeutic efficacy compared to PDT or PTT alone.

Poor light penetration could be a limitation to treat deep tumors in the field of research in PDT. A solution that has been related first by the Chen’s team (Chen and Zhang 2006) in 2006 is the use of X-rays, instead of light, combined with NPs as a new PDT modality. Since this date, other teams have developed different kinds of NPs to perform X-ray PDT. Our team (Bulin et al. 2013) synthesized terbium oxide NPs coupled to a porphyrin and showed the formation of ^1^O_2_ upon X-ray excitation. Wang et al. (2016) demonstrated the efficiency of SrAl_2_O_4_:Eu^2+^ NPs with MC540 as a Ps co-loaded in mesoporous silica in vivo with a subcutaneous tumor model or H1299 cells into the lung. The use of microwave appears also as a promising alternative as a source of excitation. The microwaves penetrate deeper than UV and visible light, and propagate through all kinds of tissues. This kind of electromagnetic wave can be useful in order to tackle the issue of small penetration of light. Only recently, microwave-induced PDT has emerged as a new and interesting phenomenon (Gu 2013). The proof of concept for this process was reported by Yao et al. using copper cysteamine NPs to destroy rat osteosarcoma cells (Yao et al. 2016). In parallel, graphitic-phase carbon nitride quantum dots have been explored by the same team (Chu et al. 2017) as a new agent for microwave-induced PDT. The singlet oxygen production under microwave irradiation was assessed and the NPs were efficient to kill cancer cells and promote tumor cell death.

Hence, this review is dedicated to bring together all the reported literature to date on the development of ZnO, TiO_2_, fullerene, and graphene NPs in the specific field of PDT and/or PTT applications.

## Zinc oxide nanoparticles (ZnO NPs)

ZnO NPs have long been discovered to have excellent physico-chemical properties as drug delivery vehicles. The Food and Drug Administration (FDA) has recognized ZnO as safe due to its lack of or very weak dark toxicity in vitro and in vivo (Hu et al. 2013).

The advantage that could be firmly associated with ZnO NPs in PDT is their ability to generate visible light upon X-ray radiation. Due to the fact that most currently available PSs absorb light at low wavelength, ZnO NP is a good candidate in improving PDT efficiency because its UV emission upon X-ray excitation more or less matches the UV absorption of most PSs, and hence has a good potential to serve as an irradiation source for PDT on deep-seated tumors. This concept is called self-lighting photodynamic therapy (SLPDT) which was first described by Chen and Zhang (2006) and updated by Sadjadpour et al. (2016).

NPs, due to their small size, have the ability to reach internal organs and tissues through small arteries, veins, and blood capillaries. In the case of ZnO NPs, the unique property described above also allows the NPs to act as a PS themselves in the presence of suitable light dose, hence opening an excellent chance of delivering PDT application to the tumor areas that are difficult to reach by conventional PSs. For the purpose of this review however, we will only focus on the application of ZnO NPs as a carrier for the delivery of second-generation PS. Three types of excitation can be applied: (a) UV-A to excite ZnO NPs, (b) UV–visible to excite PS, and (c) X-ray for fluorescence resonance energy transfer (FRET) between ZnO NPs and PS.

**Fig. 1:**
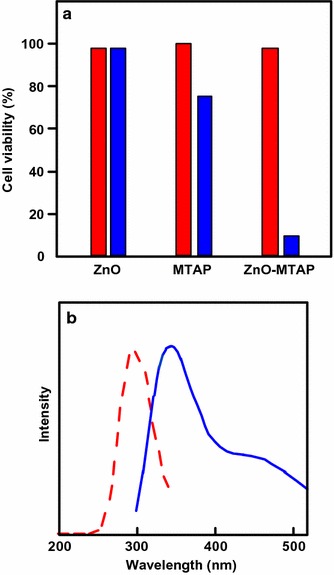
**a** Cell viability in NIH:OVCAR-3 cells exposed to either ZnO NPs alone (0.3 μM), MTAP alone (0.075 μM), or ZnO–MTAP conjugates (0.3 μM, ZnO/MTAP ≈ 4/1) under dark conditions (red) or UV illumination (light gray, 365 nm, 30 min, blue). **b** Excitation (red) and emission (blue) spectra of ZnO NPs [Taken from reference (Liu et al. 2008)]

In a work reported by Liu et al. (2008), a conjugate of ZnO–MTAP (*meso*-tetra(*o*-aminophenyl)porphyrin) was prepared. Its PDT effects were investigated on a human ovarian carcinoma cell line (NIH:OVCAR-3) and compared with ZnO NPs and MTAP as free molecules. It was shown that the conjugate has significant photocytotoxicity on the cell lines as compared to ZnO NPs or MTAP alone, in which only 10% cells were viable after UV irradiation (365 nm, 30 min) as compared to 98 and 75% for ZnO NPs and MTAP, respectively (Fig. 1). These results suggested that ZnO NP is not cytotoxic to NIH:OVCAR-3 cell lines both in dark and under UV irradiation, while the ZnO–MTAP conjugate showed a significant improvement of photocytotoxicity in the presence of light. The authors estimated 83% of energy transfer efficiency from ZnO to MTAP.

They also reported the photophysical properties of the ZnO NPs. The NPs present an excitation peak at 300 nm and two emission peaks at 345 and 445 nm, and hence the emission of ZnO NP overlaps with the absorption band of porphyrin and many other PSs. They proposed that this property could be utilized for the SLPDT concept as mentioned earlier.

**Fig. 2:**
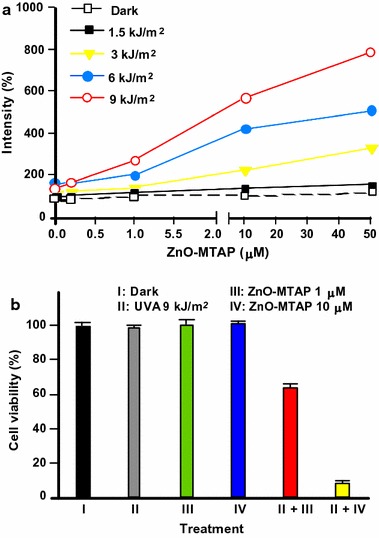
**a** ROS generation by ZnO–MTAP conjugates in the presence or absence of UV-A light. **b** OVCAR-3 cell viability (20 h later) exposed to ZnO–MTAP conjugates (1 or 10 μM) under dark or UV-A conditions (0 or 9 kJ/cm^2^ after 4-h exposure) [Taken from reference (Zhang et al. 2008)]

In a subsequent study, Zhang et al. (2008) reported singlet oxygen (^1^O_2_) production and in vitro cytotoxic effects of the water-soluble ZnO–MTAP conjugate. They used 2,7-dichlorodihydrofluorescein diacetate acetyl ester (CM-H_2_DCFDA) as a molecular probe and subsequently the fluorescence intensity was determined using a microplate reader under excitation/emission at 485/530 nm. The production of reactive oxygen species (ROS) was dependent on concentration and irradiation dose. Cell viability was estimated through MTT (methylthiazolyl tetrazolium) assay, which has a direct link to mitochondrial enzymes. A reduction in MTT assay was observed in cells which were loaded with ZnO–MTAP conjugate and subsequently irradiated, suggesting that the conjugate was photoactivated, generating ROS and leading to mitochondrial damage and hence reduced cell viability. Only 8% of cells are viable after a co-treatment of UV-A and a higher dose of ZnO–MTAP conjugate (treatment II + IV, Fig. 2).

**Fig. 3:**
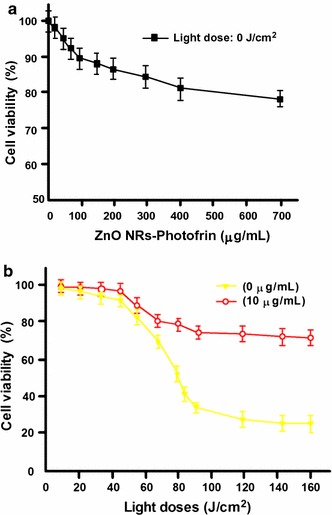
HepG2 cell viability exposed to ZnO NRs conjugated to Photofrin^®^ **a** in the absence of light and **b** in the presence of diode laser light (240 nm) [Taken from reference (Atif et al. 2011)]

ZnO nanorods (NRs) conjugated with Photofrin^®^ were investigated by Atif et al. (2011). Hepatocellular carcinoma (HepG2) cell viability was investigated upon exposure to the conjugates and UV light at 240 nm. Even in the absence of UV irradiation, the cell viability in HepG2 cells was found to decrease, although slowly, with increasing conjugate concentration administered from 0 to 800 µg/mL. Around 80% cells remained viable at the highest concentration (Fig. 3a). In the presence of UV light from diode laser light (240 nm), the white light emitted by ZnO NRs activated Photofrin^®^, which subsequently produced ROS and cell necrosis within few minutes. Hence 77% of cell viability could be observed (Fig. 3b).

The same year, Fakhar-e-Alam et al. (2011a) also reported the application of ZnO NPs conjugated with Photofrin^®^ and 5-ALA (5-aminolevulinic acid) for PDT on HepG2 cells. They observed that both conjugates of ZnO NPs showed minimal toxicity in the absence of light, but they can enhance the fluorescence in the cells due to Photofrin^®^ or protoporphyrin IX (PpIX). ZnO NPs conjugated to 5-ALA were found to better enhance the endogenous fluorescence in the HepG2 cells, as compared to ZnO NPs conjugated to Photofrin^®^. However, they reported that upon irradiation with visible light at 635 nm, no significant difference in viability of the cells treated with the two conjugates was observed as compared to ZnO NPs alone, implying the inability of the presence of PS in NPs to induce cell death in the HepG2 cells.

**Fig. 4:**
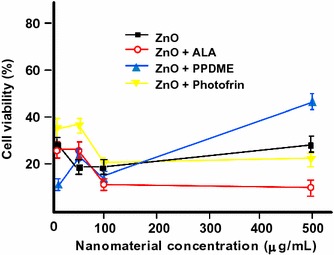
Viability of HeLa cells treated with different concentrations of ZnO NRs and their ligands with PSs [Taken from reference (Fakhar-e-Alam et al. 2011b)]

In another publication, Fakhar-e-Alam et al. (2011b) tested the cytotoxicity of ZnO NPs in the form of NRs as bare and in conjugation with different PSs which were 5-ALA, Photofrin^®^, or PPDME (protoporphyrin dimethyl ester) under irradiation at 635 nm on HeLa cells. They reported that the treatment with bare ZnO reduced cell viability by 75%, and the conjugation with PSs showed further reduction of cell viability, 90% reduction for ZnO NPs conjugated to 5-ALA and 80% reduction for ZnO NPs conjugated to Photofrin^®^. As in the case of PPDME, there was an increase of cell viability at higher conjugate concentrations as compared to the lower ones (Fig. 4). Hence, ZnO–5-ALA and ZnO–Photofrin^®^ conjugates are photocytotoxic on HeLa cells at 635 nm but are less efficient on HepG2 cells in the same conditions.

The same team (Fakhar-e-Alam et al. 2012) published a study on the effect of nanoporous ZnO NPs conjugated with Photofrin^®^ on human lung cancer cells (A549 cells). They found that upon UV irradiation ZnO–Photofrin^®^ conjugate displayed valuable reduction in cell viability as compared to Photofrin^®^ alone. Significant ROS production was observed and about 92% of cells were killed. They also established that the acceptable incubation period for PDT effect is 10–18 h with the optimal time being 12 h. They concluded that ZnO NP has a synergistic effect with Photofrin^®^ and hence a good potential for PDT application in A549 cancer cells.

Fakhar-e-Alam et al. (2014b) explored the application of ZnO NPs in PDT from different angles and application methods, in which ZnO NPs were investigated as a drug delivery vehicle for PSs. Bare ZnO NPs and ZnO NPs PEGylated PpIX were prepared as the model PS and the cell-killing effect on human muscle carcinoma RD cells was tested. In the absence of laser light, ZnO NPs at 1 mM concentration has very low cytotoxicity; 98% of RD cells are viable after a 12 h incubation period, in comparison to the ZnO NPs PEGylated PpIX in which only 85% of cells are viable at a much lower concentration, 0.2 mM. In the presence of laser light (630 nm, 80 J/cm^2^), the cytotoxicity of ZnO NPs PEGylated PpIX was very evident and the complex induced cell damage. Besides, good localization of the drug was obtained in the tumor area, showing that ZnO NPs are indeed a good drug delivery system.

D’Souza et al. (2015) studied the photophysical properties of phthalocyanines (Pcs) in the presence of ZnO NPs. They proposed that Pc adsorbed on the surface of ZnO NPs spontaneously at an average ratio of 12:1 and subsequent changes in the photophysical properties of the Pcs could be observed. The fluorescence quantum yield of Pc was lower and the fluorescence lifetime is shorter in the presence of ZnO NPs. They concluded that the presence of ZnO NPs influences the fluorescence behavior of Pcs and this could be an advantage in the application of ZnO NPs with Pcs for PDT.

**Fig. 5:**
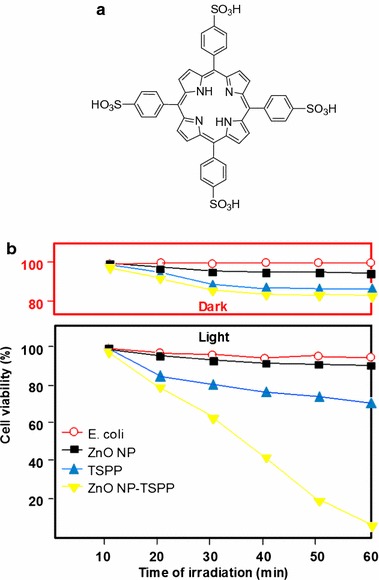
**a** Chemical structure of TSPP. **b** Antimicrobial activity of ZnO, TSPP, and ZnO NP-encapsulated TSPP on *E. coli* bacterial cells under dark and visible light irradiation [Taken from reference (Senthilkumar et al. 2013)]

Besides anticancer PDT application, PDT is also effective in antimicrobial therapy. Senthilkumar et al. (2013) investigated the action of ZnO NP-encapsulated TSPP (*meso*-tetra(4-sulfonatophenyl)porphyrin, Fig. 5a) for antipathogen PDT. It was already known that ZnO NP has the ability to inhibit the growth of different pathogenic bacteria under normal visible light. TSPP which is an anionic PS has low photoinactivation of Gram-negative bacteria such as *E. coli*. The encapsulation of TSPP in ZnO NPs was believed to be able to increase the photoinactivation of *E. coli* through increased cellular delivery and improved ^1^O_2_ production. They indeed showed that under visible light (400–800 nm), ZnO NP-encapsulated TSPP has significantly higher antibacterial activity as compared to TSPP or ZnO NPs alone (98% activity as compared to 30%). The activity of TSPP alone was negligible on *E. coli* under visible light (Fig. 5b).

**Fig. 6:**
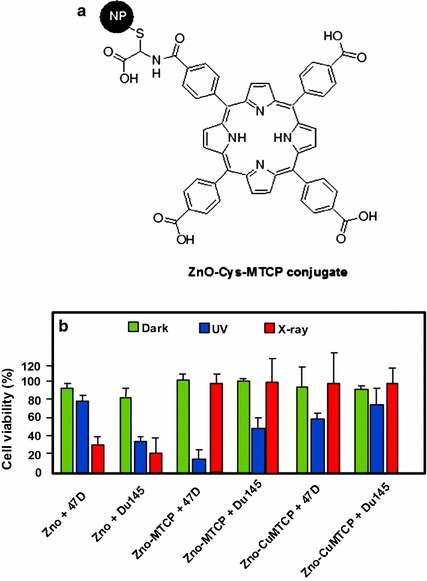
**a** Chemical structure of ZnO–MTCP conjugate. **b** Viability of T-47D and DU145 cells incubated with ZnO, ZnO–MTCP, and ZnO–CuMTCP at 60 μM concentration under dark condition, UV irradiation (UV-A/B, 100 μW/cm^2^ for 3 min), and X-ray irradiation (0.94 Gy, 30 s) [Taken from reference (Sadjadpour et al. 2016)]

Sadjadpour et al. (2016) studied the synthesis and conjugation of ZnO NPs with two different porphyrins, *meso*-tetra(4-carboxyphenyl)porphyrin (MTCP) and CuMTCP (Fig. 6a), and their PDT effects on prostate DU145 and breast T-47D cancer cells. The conjugation was performed by first coating the surface of ZnO NPs with l-cysteine and subsequently it was conjugated with the porphyrin. The fluorescence intensity of ZnO NP-coated l-cysteine was mostly quenched at 370 nm after conjugation with porphyrin and a new peak corresponding to the energy transfer between ZnO NP-coated l-cysteine and the conjugated porphyrin molecules appeared. The T-47D cells were more resistant towards PDT treatment by the conjugates as compared to the DU145 cells, and this could be due to the presence of a defense mechanism against NPs uptake by the T-47D cells. Between the two conjugates (ZnO–MTCP and ZnO–CuMTCP), ZnO–MTCP showed higher photocytotoxicity under UV irradiation (UV-A/B, 100 μW/cm^2^ for 3 min) towards the two selected cell lines as compared to ZnO NPs alone, which indicated an efficient FRET between ZnO and PS (Fig. 6b). In contrast, under X-ray irradiation (0.94 Gy, 30 s), ZnO NPs alone gave significant cytotoxicity on both T-47D and DU145 cell lines, while no cytotoxic effect was observed when the cells were treated with the two conjugates (Fig. 6b). The authors are of the opinion that this may be due to a lack of FRET between ZnO and PS in the conjugated compounds.

It is also important to note that although ZnO NP is regarded as having the necessary biosafety and biocompatibility profiles and recently its toxicology profile has gained more attention. The toxicity of ZnO NPs is regarded due to its dispersibility. The solubility of ZnO in the extracellular region could lead to an increase in the intracellular Zn^2+^ level and this might subsequently induce cytotoxicity through certain mechanisms that are still unclear (Pandurangan and Kim 2015). Interestingly, research has shown that surface modification techniques such as PEGylation could reduce its cytotoxicity by reducing its cellular intake (Luo et al. 2014). Surface coating with polymers has also been shown to reduce cytotoxicity as reported by Osmond-McLeod et al. (2014). Another technique called “ron-doping” was also found to have a reduction effect on the cytotoxicity of this particle, example being on lung toxicity as reported in several papers (Xia et al. 2011; Cho et al. 2012). Nevertheless, more studies are needed to determine specifically the mechanism of toxicity and the exact safety conditions of ZnO application as NPs.

In summary, ZnO is a semiconductor material already used for water treatment. More recently, ZnO NPs act as a good candidate for PDT or antimicrobial therapy. Thanks to its small size and the various forms that they can adopt (nanoparticles or nanorods), ZnO–PS systems are good drug delivery systems. Conjugated ZnO–PS systems possess great photophysical properties to obtain a good PDT efficiency and have also evidenced a good ROS production (^1^O_2_ and other radicals). Biologically, ZnO–PS systems present good phototoxicity and, more recently, their toxicity have been investigated and some studies showed that the solubility of ZnO in the extracellular region could lead to an increase in the intracellular Zn^2+^ level inducing cytotoxicity. Other studies are led to determine the safety conditions of ZnO NPs’ utilization. Table 1 below summarized the data available from the literature on the application of ZnO NPs in PDT.

**Table 1:** Application of ZnO NPs in PDT

Type of NPs (size, nm)	PS (amount)	NPs–PS interactions	Irradiation conditions	Type of ROS	Cancer cell line		Refs.
					In vitro	In vivo	
NPs (5)	MTAP (4/ZnO)	Conjugated by coupling reaction	UV light 365 nm, 0.51 W/cm^2^, 30 min	nd	NIH:OVCAR-3	–	Liu et al. (2008)
NPs (5)	MTAP (4/ZnO)	Conjugated by coupling reaction	UV-A lamp 365 nm, 0.50 W/cm^2^, 30–120 min	ROS	NIH:OVCAR-3	–	Zhang et al. (2008)
Nanorods (130–150)	Photofrin and 5-ALA (nd)	Conjugated	Diode laser 630 nm along with UV light of 240 nm, 0–160 J/cm^2^, 0–20 min	ROS	HepG2	SD rats	Atif et al. (2011)
NPs (80–120)	Photofrin and 5-ALA (nd)	Conjugated	Diode laser 635 nm, 80 J/cm^2^	nd	HepG2	–	Fakhar-e-Alam et al. (2011a)
Nanorods (150–200)	Photofrin, 5-ALA and PPDME (nd)	Conjugated	Diode laser 635 nm, 30 J/cm^2^, 6.5 min	ROS	HeLa	–	Fakhar-e-Alam et al. (2011b)
Nanoporous (200–600)	Photofrin (nd)	Conjugated	Diode laser, 80 J/cm^2^	ROS	RD	–	Fakhar-e-Alam et al. (2012)
NPs (35)	PpIX (nd)	Encapsulated	Laser 630 nm, 80 J/cm^2^	ROS	A549	–	Fakhar-e-Alam et al. (2014b)
Nanowires (150–170)	PpXI (nd)	Conjugated	UV-A illumination, light dose 10 J/cm^2^	ROS	FM55P AG01518	–	Fakhar-e-Alam et al. (2014a)
NPs (3–26)	MTCP and CuMTCP (nd)	Conjugated by coupling reaction	UV-A/B (100 μW/cm^2^, 3 min) or X-ray (0.94 Gy, 30 s) irradiation	ROS	DU145 T-47D	–	Sadjadpour et al. (2016)
Nanohexagons Nanorods (25–90)	ZnPc ZnTMAAPc ZnTMPAPc (OH)AlPcSmix (Pc: ZnO NPs, 12:1)	Conjugated by coupling reaction	669–690 nm	nd	‒	–	D’Souza et al. (2015)
NPs (25–40)	TSPP (nd)	Encapsulated	Visible light irradiation	^1^O_2_	*S. aureus* *E. coli* KCCM 12234 KCCM 11256	–	Senthilkumar et al. (2013)

*NPs* nanoparticles, *PS* photosensitizer, *ROS* reactive oxygen species, *nd* not disclosed, *MTAP meso*-tetra(*o*-aminophenyl)porphyrin, *UV* ultraviolet, *5*-*ALA* 5-aminolevulinic acid, *SD* Sprague Dawley, *PPDME* protoporphyrin dimethyl ester, *PpIX* protoporphyrin IX, *MTCP meso*-tetra(4-carboxyphenyl)porphyrin, *ZnPc* zinc phthalocyanine, *ZnTMAAPc* 2,(3),9(10),16(17),23(24)-tetrakis-(mercaptoacetic acid phthalocyaninato) zinc(II), *ZnTMPAPc* 2,(3),9(10),16(17),23(24)-tetrakis-(mercaptopropanoic acid phthalocyaninato) zinc(II), *(OH)AlPcSmix* mixture of the di-, tri-, and tetra-sulfonated phthalocyanine derivatives, *ZnO* zinc oxide, *TSPP meso*-tetra(4-sulfonatophenyl)porphyrin

## Titanium dioxide nanoparticles (TiO_2_ NPs)

Titanium dioxide fine particles (TiO_2_ FPs) have long been manufactured and used worldwide for different applications. In the past, they were considered to have very low toxicity. However, a finding reported in 1985 has provided a new perspective on its safety. It was found that a chronic exposure of this FPs in mice at a high concentration of 250 mg/m^3^ for 2 years (6 h/day for 5 days/week) could lead to bronchioloalveolar adenomas and cystic keratinizing squamous cell carcinomas (Lee et al. 1985). However, the detected tumors were different from the common human lung tumors and no metastases could be observed. In the opinion of the researchers, this finding has no biological relevance for human and it is also possible that the tumor was due to overloaded TiO_2_ FPs instead of specific carcinogenicity of the particles (Lee et al. 1985; Shi et al. 2013).

TiO_2_ NPs are found to have different physico-chemical properties which may lead to changes in their bioactivity. This NP has been used widely in industrial and consumer products due to their strong catalytic activity (Shi et al. 2013). TiO_2_ is also known as a wide-band gap semiconductor and is photoactive in the presence of UV light against microorganisms and cancel cells. Efforts have been made to use TiO_2_ in the form of NP as a support in PDT, by grafting PSs onto the TiO_2_ NP surface. This grafting enables the use of visible light, instead of UV light for TiO_2_ alone, in the activation of TiO_2_ NP-conjugated PS (Jia and Jia 2012). Several studies have shown promising results in the application of these TiO_2_ NPs conjugated to PS and will be described below.

**Fig. 7:**
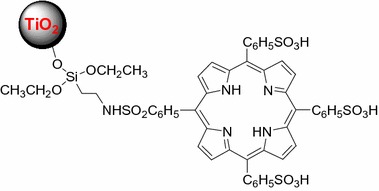
Chemical structure of TiO_2_–Sil–TSPP conjugate [Taken from reference (Ion and Brezoi 2005b)]

**Fig. 8:**
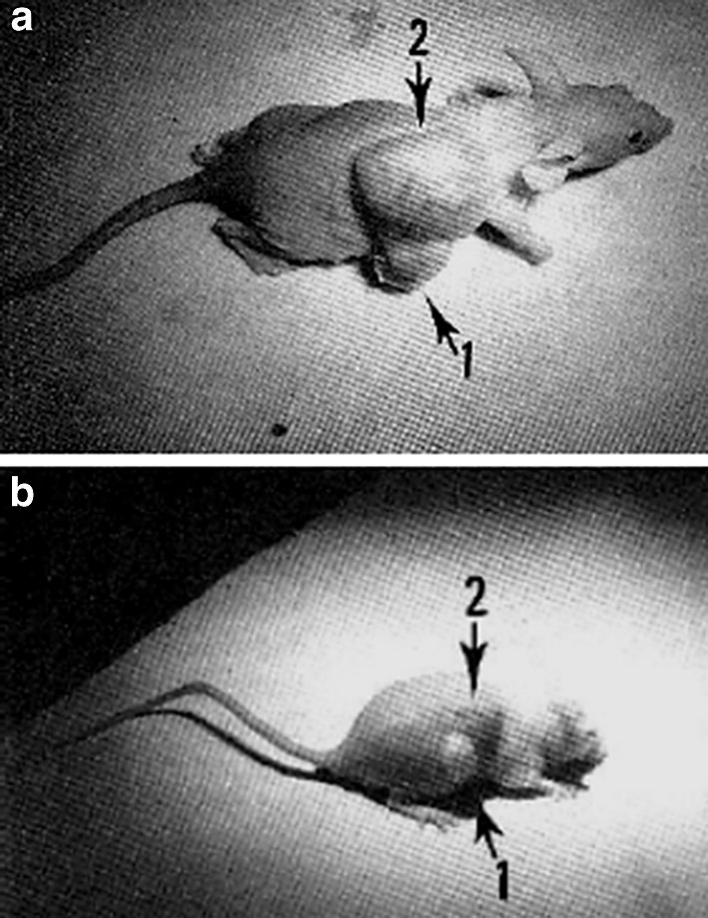
Effect of laser before (**a**) and after (**b**) illumination (no detail given) [Reused with permission from reference (Ion 2004)]

In the early 2000s, Ion and Brezoi (Ion 2004; Ion and Brezoi 2005a) described the synthesis of a new conjugate TiO_2_–Sil–TSPP consisting of a coupling between *meso*-tetra(4-sulfonatophenyl)porphyrin (TSPP) bearing a silane arm (Sil) with TiO_2_ NPs (Fig. 7). Electronic absorption spectra confirmed the binding of Sil–TSPP to the TiO_2_ NPs. Atomic force microscopy (AFM) images taken have enabled the determination of the conjugated NP size which was around 39 nm. The in vivo study of this TiO_2_–Sil–TSPP conjugate on an animal model (mice with implanted cancer cells under skin) showed that the conjugate inhibited tumor growth after laser irradiation (Fig. 8). No further details are given.

Lopez et al. (2010) synthesized via the sol–gel method a TiO_2_–ZnPc conjugate by the incorporation of ZnPc into the porous network of TiO_2_ NPs. The different techniques (IR, UV–Vis, Raman spectroscopies) proved that Pc and NPs were linked covalently by the *N*-pyrrole. The photosensitizing effects of ZnPc, TiO_2_ NPs, and TiO_2_–ZnPc conjugate have been studied against four mammalian cells and on two forms of *Leishmania* parasites (Table 2). Under light irradiation (670 nm), TiO_2_ NPs were not phototoxic to the cells, as expected. In the same conditions, the treatment with ZnPc was photoactive for all the mammalian cells and a higher phototoxic effect was observed using 597–752 nm irradiation compared to 670 nm irradiation. Nevertheless, the activity against mammalian cells of TiO_2_–ZnPc conjugate was lower than that of the ZnPc alone. TiO_2_–ZnPc conjugate had no phototoxicity for *Leishmania* parasites. The internalization of TiO_2_–ZnPc conjugate by the cells was lower than that for ZnPc alone. The localization of TiO_2_–ZnPc conjugate and ZnPc alone was observed in mitochondrial cytoplasm. Finally, the authors concluded that the TiO_2_–ZnPc conjugate could be a potential PS for PDT treatment.

**Table 2:** Photoactivity of ZnPc and ZnPc–TiO_2_ on *Leishmania* promastigotes and different mammalian cell lines

	Fluency (J/cm^2^)	IC_50_ (μM)		CC_50_ (μM)			
		*Leishmania* parasites		Mammalian cell lines			
		*Chagasi*	*Panamensis*	HDFs	THP-1	HepG2	Vero
ZnPc	0	>15	14.76	9.21	7.74	10.70	0.78
	2.5^a^	12.86	6.63	1.08	0.14	0.28	0.24
	2.5^b^	0.19	0.39	0.15	0.02	0.035	0.09
	10^a^	5.63	5.63	0.05	0.17	0.086	0.038
ZnPc–TiO_2_	0	> 10	> 10	> 10	> 10	> 10	0.087
	2.5^a^	> 10	> 10	5.51	7.45	> 10	0.079
	2.5^b^	> 10	> 10	3.54	0.28	0.43	0.023
	10^a^	> 10	> 10	nd	2.00	5.50	0.001

*ZnPc* zinc phthalocyanine, *IC*_*50*_ concentration that induces 50% of parasite inhibition, *CC*_*50*_ cytotoxic concentration that induces 50% of cell death, *HDFs* human-derived fibroblasts, *THP-1* human macrophages, *HepG2* human heptocellular carcinoma cells, *nd* not disclosed

^a^Laser light irradiation (670 nm)

^b^Biological photoreactor irradiation (597–752 nm)

**Fig. 9:**
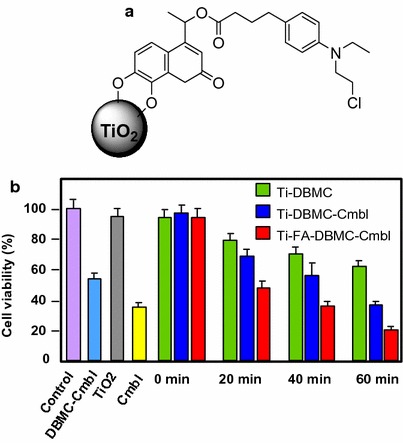
**a** Chemical structure of Ti–DBMC–Cmbl NPs. **b** Viability of MDA-MB-231 cells treated with Ti–DMC, Ti–DBMC–Cmbl NPs, and Ti–FA–DBMC–Cmbl NPs (250 μg/mL) [Taken from reference (Gangopadhyay et al. 2015)]

Gangopadhyay et al. (2015) described the use of TiO_2_ NPs loaded with 7,8-dihydroxy-4-bromomethylcoumarin–chlorambucil (Ti–DBMC–Cmbl NPs, Fig. 9a) as a targeted combination therapeutic system for MDA-MB-231 breast cancer cells. This conjugate combines the PDT via the coumarin chromophore and the chemotherapy by the chlorambucil drug. Spectroscopic characterizations (IR, UV–Vis, and fluorescence) confirmed the binding of DBMC–Cmbl to TiO_2_ NPs. The conjugate possess three absorption peaks at 250, 330, and 500 nm. The authors found that the Ti–DBMC–Cmbl NPs have a size of 164.18 nm with good PDT efficiency and a ^1^O_2_ quantum yield of 0.29 when excited at 425 nm. The in vitro studies on MDA-MB-231 breast cancer cells showed a good uptake of the conjugate in tumor cells, an inhibited proliferation, and a significant induction of apoptosis (Fig. 9b). Furthermore, for better tumor targeting, they functionalized the Ti–DBMC–Cmbl NPs with folic acid (FA) to target folic acid receptor which is overexpressed on the surface of certain cancer cells (Reddy et al. 2005; Sega and Low 2008). The resulting Ti–FA–DBMC–Cmbl NPs appeared much more efficient than Ti–DBMC–Cmbl NPs after 1 h of irradiation (≥ 410 nm) and the authors observed only ~ 19% cell viability compared to ~ 35% cell viability in the case of the conjugate without FA (Fig. 9b).

**Fig. 10:**
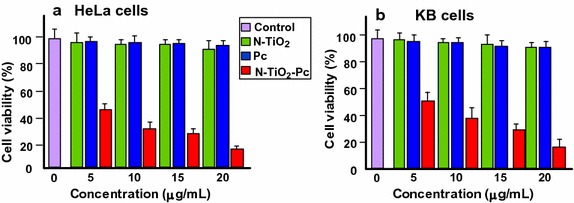
Comparative Viability of **a** HeLa cells and **b** KB cells treated with Pc, N–TiO_2_ NPs, and N–TiO_2_–Pc NPs (250 μg/mL) [Taken from reference (Pan et al. 2015)]

To improve the cellular uptake of aluminum phthalocyanine chloride tetrasulfonate (AlPcS_4_, named Pc), Pan et al. (2015) used nitrogen-doped TiO_2_ NPs (N–TiO_2_ NPs) to carrier Pc. They obtained the N–TiO_2_–Pc conjugate by a two-step synthesis: the first step consisted in a silanization reaction of N–TiO_2_ with 3-aminopropyltriethoxysilane (APTES) to obtain N–TiO_2_–NH_2_ followed by the conjugation of Pc. Transmission electron microscopy (TEM) analysis showed that the size was around 25–40 nm for the conjugate. The authors compared the absorption spectra of Pc alone and N–TiO_2_–Pc conjugate, which expands from 400 to 800 nm, resulting in a 2.6 times better production of ROS under visible light irradiation compared to Pc alone. In vitro studies on HeLa cells (Fig. 10a) and KB cells (Fig. 10b) showed that the cellular uptake of the conjugate was enhanced 6.0 times than Pc alone and its phototoxicity was low. Confocal microscopy allowed to detect N–TiO_2_–Pc conjugate in the nucleus area. The photokilling effect was also evaluated (Fig. 10) and the results suggested that N–TiO_2_–Pc conjugate could be an excellent candidate as a PS in PDT.

It is well known that TSPP (see Fig. 5a) possesses adverse effects hampering its potential use in PDT. TSPP enters the cell via endocytosis and localizes in cellular organelle (lysosomes, endosomes, and endoplasmic reticulum) where it interferes with cellular signal pathways, thus producing apoptosis or necrosis (Høgset et al. 2004; Berg et al. 2011). Rehman et al. (2015, 2016b) studied the protective effect of TiO_2_ nanowhiskers (TiO_2_ NWs)–TSPP complex in vitro and in vivo. TSPP–TiO_2_ NWs were prepared by mixing TSPP and TiO_2_ together. Various concentrations of TiO_2_ NWs, TSPP, and TiO_2_–TSPP NWs were injected into rats belonging to four different groups (the fourth group is the control). Histopathology, complete blood cell count (CBC), and fluorescent microscopy were used to evaluate the toxic effects on excretory and circulatory system. The CBC, histopathology, and fluorescent microscopic studies also showed that low concentration TSPP–TiO_2_ NWs were more secure. The in vitro cytotoxicity was evaluated and a maximum viability was showed for illuminated TSPP–TiO_2_ NWs group. To conclude, the authors proposed that the use of these TSPP–TiO_2_ NWs would be adapted for the PDT and bioimaging of cancer or other diseases.

**Fig. 11:**
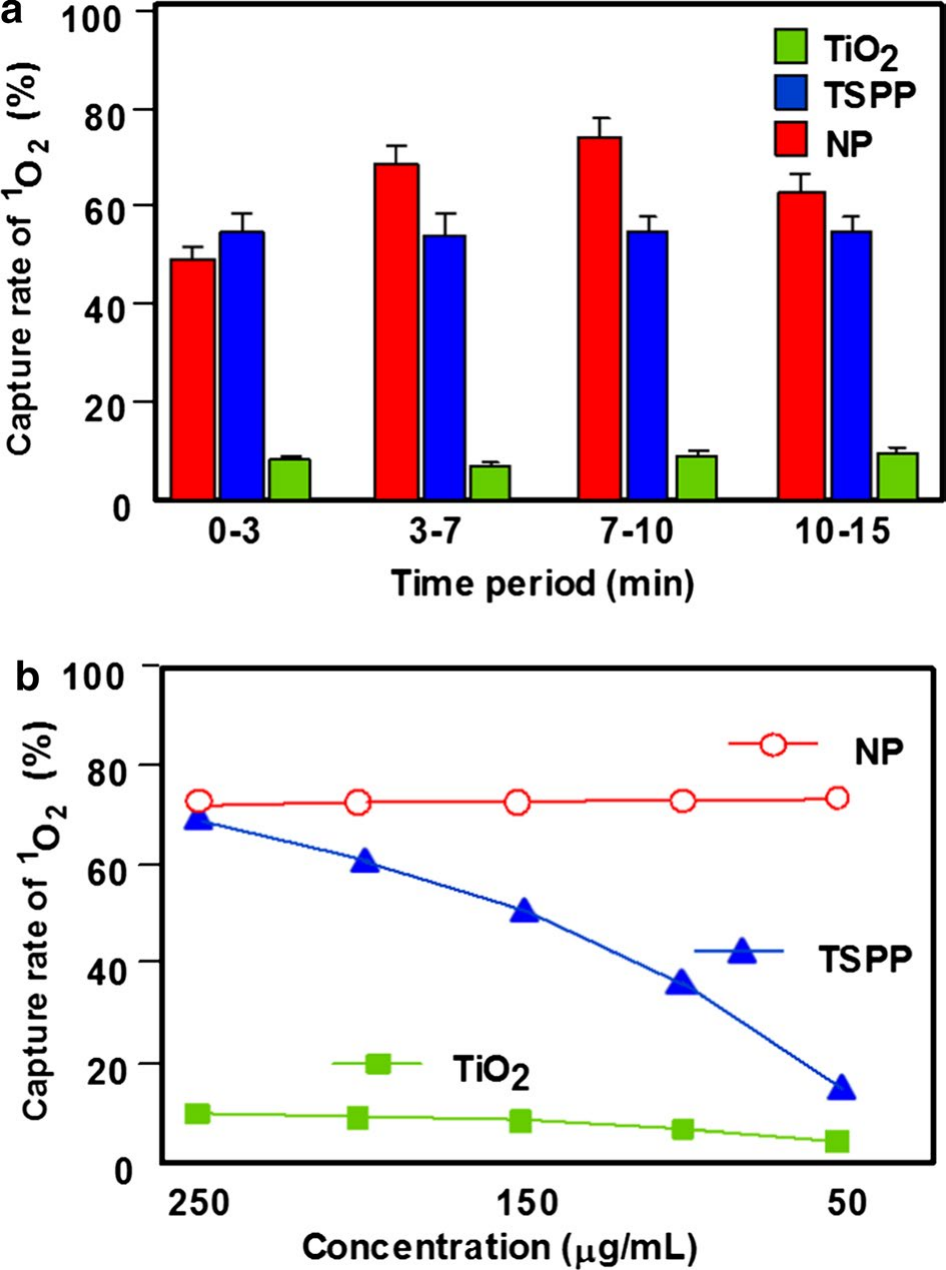
^1^O_2_ quantum yield determination **a** at different time points and **b** at various concentrations for TSSP–TiO_2_ NPs and TSPP and TiO_2_ alone. The ^1^O_2_ quantum yields correspond to the 1,3-diphenylisobenzofuran (DPBF) capture rate of ^1^O_2_ [Taken from references (Zhao et al. 2015, 2016)]

The same team (Zhao et al. 2015, 2016) was interested in the target cellular bioimaging and treatment of rheumatoid arthritis using TiO_2_–TSSP NPs, which were obtained by mixing a solution of TSPP in phosphate buffer saline (PBS) to a suspension of TiO_2_ in an acetic acid/sodium acetate buffer. The TiO_2_–TSSP NPs were characterized by UV–Vis, fluorescence, and IR spectroscopies and zeta potential measurement showing that the porphyrin is covalently linked to TiO_2_ NPs by the NH-pyrrole. The TSPP loading capacity on TiO_2_ NPs was about 17.4 wt%. The scanning electron microscopy (SEM) images showed that the TSSP–TiO_2_ NPs were agglomerated, whereas TiO_2_ alone had a 30 nm diameter. Figure 11 shows the capability of NPs to produce enough ^1^O_2_ and guarantee a good PDT effect. The in vitro studies on the HSC (human rheumatoid arthritis synovial fibroblast cells) and the RSC (murine rheumatoid arthritis synovial fibroblast cells) showed that the TSSP–TiO_2_ NPs were less cytotoxic than the TSSP alone and demonstrated a good inhibition of the cellular growth of the synovial fibroblast. These NPs act as good candidates for the theranostic biomarkers for rheumatoid arthritis.

**Fig. 12:**
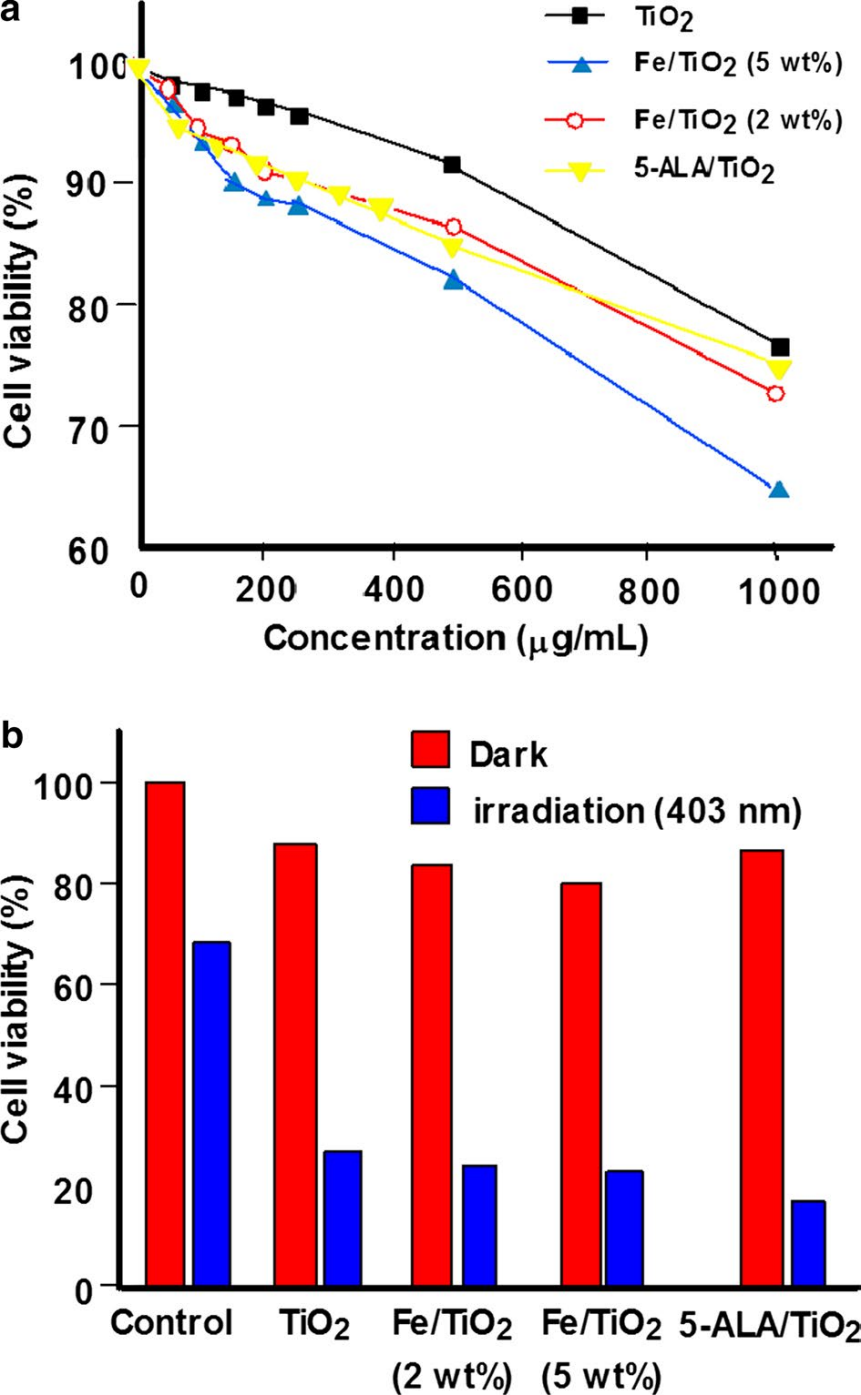
**a** Dark toxicity of TiO_2_ and modified TiO_2_ NPs on HL60 cells at different concentrations. **b** Viability of HL60 cells treated with TiO_2_ and modified TiO_2_ NPs upon irradiation (1 h, 403 nm, 5 mW/cm^2^, 18 J/cm^2^) [Taken from reference (Lu et al. 2015)]

**Fig. 13:**
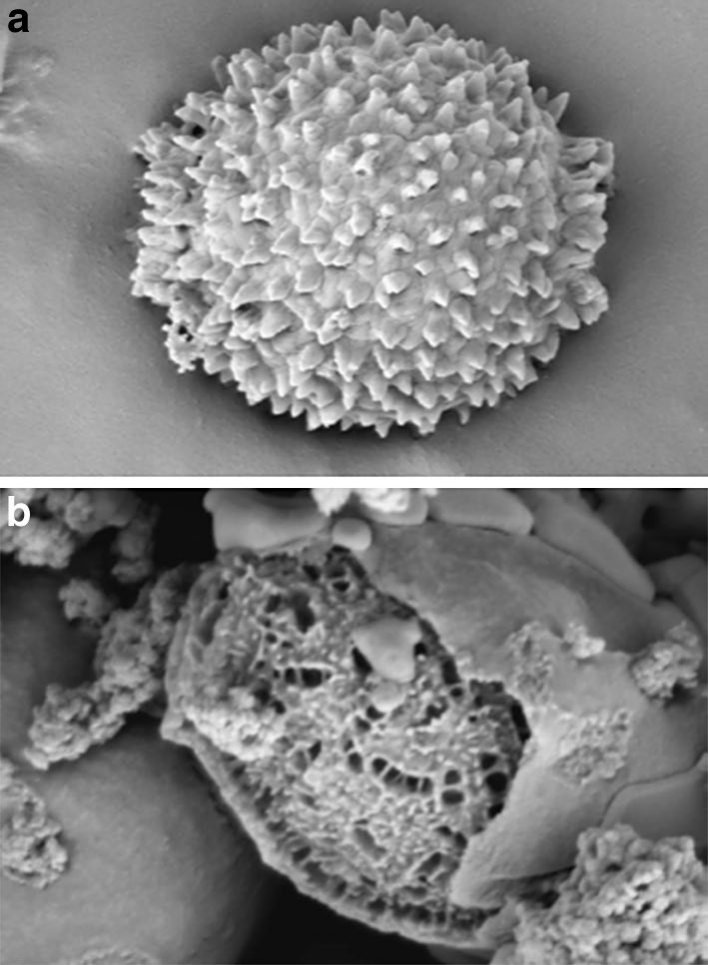
Ultrastructural morphology of HL60 cells. **a** Normal cultured cells. **b** PDT-treated cells cultured with 5-ALA/TiO_2_ NPs [Reused with permission from reference (Lu et al. 2015)]

Lu et al. (2015) studied the PDT effect of Fe- and 5-ALA-modified TiO_2_ NPs, i.e., Fe/TiO_2_ and 5-ALA/TiO_2_ NPs. Fe/TiO_2_ (2 wt%) and Fe/TiO_2_ (5 wt%) NPs were synthesized by precipitation method, while 5-ALA/TiO_2_ NPs were synthesized by ultrasonic method. All the modified TiO_2_ NPs were characterized by X-ray diffraction and UV–Vis spectroscopy. The characteristic peaks at 1430 and 1730 cm^−1^ observed on FTIR (Fourier transform infrared) spectra of 5-ALA/TiO_2_ NPs proved that 5-ALA was covalently bound to TiO_2_ NPs by an ester link. All the modified TiO_2_ NPs possess an enhanced absorption in the visible light region. Figure 12 presents the dark toxicity of modified TiO_2_ NPs to HL60 cells at different concentrations (Fig. 12a). 5-ALA/TiO_2_ NPs were less toxic to HL60 cells than Fe/TiO_2_ NPs, but more than that of TiO_2_ NPs alone. The PDT effect of modified TiO_2_ NPs was evaluated on HL60 cells after 1-h light exposure at 403 nm. Cell viability with 5-ALA/TiO_2_, Fe/TiO_2_ (2 wt%), and Fe/TiO_2_ (5 wt%) was 19.4, 28.4, and 28.0%, respectively (Fig. 12b). 5-ALA/TiO_2_ NPs present a promising PDT effect as assessed by the ultrastructural morphology of HL60 cells before and after PDT treatment (Fig. 13).

In summary, TiO_2_ is a semiconductor material extensively used in many domains including photocatalytic water treatment, solar cells, sterilization, and more recently anticancer therapy. It embraces a wide range of advantageous properties such as low cost, availability, and biological and chemical inertness. TiO_2_ can produce a significant cytotoxic effect under UV illumination, accompanied with minor dark toxicity, high stability, and good biocompatibility in vitro and in vivo. However, TiO_2_ has a UV-limited photoresponse due to its wide band gap. The electronic properties of TiO_2_ can be easily tuned by linking a PS to its surface. The PS can be either adsorbed or grafted covalently to the surface of TiO_2_ NPs. The formed TiO_2_–PS system induces an extension of the absorption profile of TiO_2_ enabling the use of the visible light for different applications. TiO_2_, in its nano-metric scale, possesses an appropriate size enabling its use as a carrier of PS to enhance the latter’s uptake into the cells, such as Pc. In addition, conjugated TiO_2_–PS system has evidenced a good ROS production (^1^O_2_ and other radicals). TiO_2_–PS systems can also serve in the cellular bioimaging. Despite all those good properties, such systems suffer from agglomeration and dispersion issues. Table 3 below summarized the data available on the application of TiO_2_ NPs grafted with or encapsulating PSs in PDT.

**Table 3:** Application of TiO_2_ NPs in PDT

Type of NPs (size, nm)	PS (amount)	NPs–PS interactions	Irradiation conditions	Type of ROS	Cancer cell line		Refs.
					In vitro	In vivo	
NPs (32–37)	TSPP (1/TiO_2_)	Conjugated via a silyl linker	Laser irradiation	^1^O_2_	‒	Skin tumors from mice	Ion and Brezoi (2005b); Ion (2004)
NPs (nd)	ZnPc (nd)	Encapsulated	Laser light 670 nm, 2.5 or 10 J/cm^2^, or biological photoreactor 597–752 nm, 2.5 J/cm^2^	^1^O_2_	THP-1 HepG2 Vero *L. chagasi* *L. panamensis*	‒	Lopez et al. (2010)
NPs (164.2)	DBMC (63.3 μg/TiO_2_)	Conjugated via an ether linker	UV–visible (≥ 410 nm), 0–60 min	^1^O_2_	MDA-MB-231	‒	Gangopadhyay et al. (2015)
NPs (25–40)	AlPcS_4_ (nd)	Electrostatic attraction	150-W Xe lamp (420–800 nm), 15 J/cm^2^	ROS	HeLa KB	‒	(Pan et al. 2015)
NPs (nd)	5-ALA (molar mass ratio 5-ALA:TiO_2_, 2:1)	H-bonds	403 ± 6 nm, 5 mW/cm^2^, 18 J/cm^2^, 60 min	nd	HL60	‒	Lu et al. (2015)
Nanowhiskers (> 100)	TSPP (15.7 wt%)	Physical absorption	Visible light 500–550 nm, 30 min	ROS ^1^O_2_	Fibroblast cells from RA joint of SD rats	Male SD strain rats and DBA-1 mice with RA disease	Zhao et al. (2015)
Nanowhiskers (30)	TSPP (17.4 wt%)	Charge transfer	Green light 500–550 nm, 5 mW/dm^2^, 10 min	^1^O_2_	HSC RSC	‒	Zhao et al. (2016)
Nanowhiskers (nd)	TSPP (nd)	Ionic bonding	Visible LED light (500–550 nm), 5 mW/dm^2^, 5 min for in vitro and 60 min for in vivo	ROS ^1^O_2_	RA infected BMS cells	BMS cells from the RA infected murine models	Rehman et al. (2015)
Nanowhiskers (nd)	TSPP (concentration TiO_2_:TSPP, 6:1)	Solution mixture	Green light 500–550 nm, 60 min	ROS ^1^O_2_	Fibroblast primary cells from SD rats	Male SD rats	Rehman et al. (2016b)
Nanowhiskers (nd)	TSPP (concentration TiO_2_:TSPP, 10:1)	Absorption	Visible light 500–550 nm, 60 min	ROS ^1^O_2_	‒	Diabetes mellitus murine model (type I and type II)	Rehman et al. (2016a)

*NPs* nanoparticles, *PS* photosensitizer, *ROS* reactive oxygen species, *nd* not disclosed, *TSPP meso*-tetra(4-sulfonatophenyl)porphyrin, *ZnPc* zinc phthalocyanine, *DBMC* 7,8-dihydroxy-4-bromomethylcoumarin, *UV* ultraviolet, *AlPcS*_*4*_ aluminum phthalocyanine chloride tetrasulfonate, *5*-*ALA* 5-aminolevulinic acid, *SD* Sprague Dawley

## Fullerene

Fullerene C_60_ has been evidenced by Kroto et al. (1985) who were awarded the 1996 Nobel Prize in Chemistry for this important discovery. From that point, this redox-active chromophore and its analogues were thoroughly studied for their electron and energy transfer ability to form artificial photosynthetic systems (Martin et al. 1998). Thus, a large number of intermolecular C_60_ charge transfer dyads were described either with electron donor molecules such as ferrocenes (Crane et al. 1992), cobaltocenes (Stinchcombe et al. 1993), or polymers (Zhang et al. 2014). In addition, numerous covalently linked C_60_–Donor dyads have been synthesized in several ways (Hirsch 1995), i.e., 1,3-dipolar cycloaddition (Maggini et al. 1993; Meier and Poplawska 1996), Diels–Alder (Belik et al. 1993), or Bingel–Hirsch (Bingel 1993; Cho et al. 2014) reactions.

Functionalized fullerenes alone can be considered as PSs usable for the medical applications as, for example, the treatment of mice infected by Gram-negative bacteria (Sharma et al. 2011; Huang et al. 2014). PDT applications have also been investigated and various cancers such as metastatic cancer in peritoneal cavity were studied. Tokuyama et al. (1993) were the first to show that fullerenes substituted by carboxylic acids could be phototoxic to HeLa cells. In the same way, it has been shown that the action of pristine C_60_ and light could be used for the treatment of Ehrlich carcinoma cells or infected thymocyte eradication in rat (Burlaka et al. 2004). Currently, research focuses more on the treatment of pathogenic infections by substituted fullerenes in the presence of light than on light-mediated PDTs of cancers and only few research articles have been submitted in the two last years, the most important being cited in the following references (Wang et al. 2014; Shi et al. 2014, 2016; Li et al. 2015b; Liu et al. 2015b; Yu et al. 2016).

As already mentioned, the subject of this review concerns the use of dyads in which a PS (electron donor) and a fullerene (electron attractor) can give rise to a PDT effect mainly due to light-induced electron transfer. An interesting review entitled “Fullerene–porphyrin nanostructures in photodynamic therapy” has been published by Constantin et al. (2010).

**Fig. 14:**
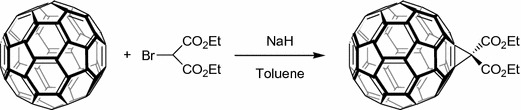
Example of malonic functionalization of fullerene

One general protocol to functionalize a fullerene is the Bingel–Hirsch reaction (Bingel 1993) which is a two-step cyclopropanation by a Michael addition of a α-halocarbanion followed by the expulsion of the halogen and subsequent malonate formation (Fig. 14).

This malonic functionalization of fullerene has been widely studied to design new molecules usable for PDT application. However, it is known that [C_60_] fullerene malonic acid derivatives (MA–C_60_) can induce damages to cytoplasmic and mitochondrial membranes (Yang et al. 2007).

**Fig. 15:**
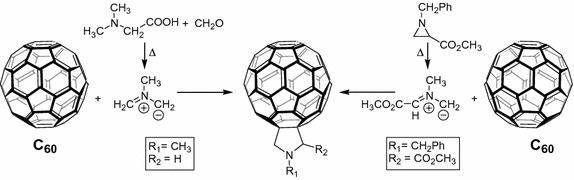
Substitution of fullerene by Prato reaction

The other classical protocol to substitute a fullerene is the Prato reaction (Maggini et al. 1993) which can proceed in two ways involving the addition of azomethine ylide to fullerene (Fig. 15).

**Fig. 16:**
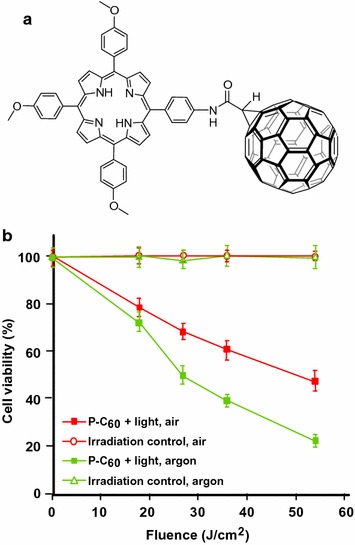
**a** Chemical structure of free base P–C_60_ dyad. **b** Inactivation of Hep-2 cells irradiated with visible light (0–54 J/cm^2^) in atmosphere of air or argon (1 μM of P–C_60_, incubation time 24 h) [Taken from references (Alvarez et al. 2006, 2009)]

Durantini’s group (Milanesio et al. 2002) designed 5-(4-amidophenyl)-10,15,20-tris(4-methoxylphenyl)porphyrin–fullerene dyad (P–C_60_) free base or metallated by Zn(II) (Fig. 16a). The synthesis involved the condensation of 1,2-dihydro-1,2-methanofullerene[60]-61-carboxylic acid (Fungo et al. 2001) and an aminoporphyrin (Fungo et al. 2000). Compared to porphyrin alone, the P–C_60_ dyads exhibited a lower emission than that of the porphyrin alone, resulting from quenching of the fullerene entity (Milanesio et al. 2005). ^1^O_2_ production quantum yield (*Φ*_Δ_) was dependent on the polarity of the solvent and *Φ*_Δ_ diminished considerably in DMF (*Φ*_Δ_ = 0.18) vs toluene (*Φ*_Δ_ = 0.80). For the in vitro studies on Hep-2 human larynx carcinoma cell line, the dyads were added from a liposomal solution due to their low solubility in PBS. Only the free base porphyrin P–C_60_ exhibited interesting properties (Alvarez et al. 2006) and was further studied. The P–C_60_ uptake occurred at a concentration of < 1.5 nM/10^6^ cells after 5–8 h. After incubation with P–C_60_ (1 μM) and irradiation at a wavelength range between 350 and 800 nm and at 54 J/cm^2^, the cell survival was about 20 and 50% under the atmosphere of air or argon, respectively (Alvarez et al. 2006, 2009) (Fig. 16b).

**Fig. 17:**
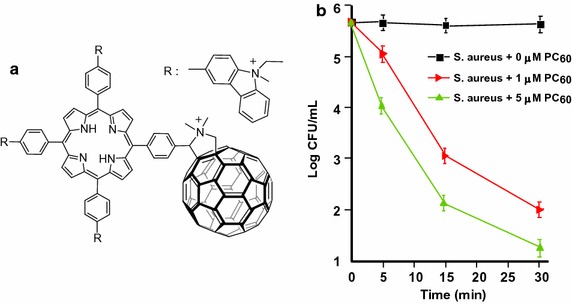
**a** Chemical structure of P–C_60_ dyad. **b** Photoinactivation of *S. aureus* by P–C_60_ fullerene at different concentrations and at a wavelength range between 350 and 800 nm [Taken from reference (Belen Ballatore et al. 2014)]

The same group (Belen Ballatore et al. 2014) described the synthesis of a similar porphyrin–fullerene dyad P–C_60_ (Fig. 17a) via a Prato reaction in which a *N*-ethylcarbazole moiety was introduced at the *meso* positions. This dyad was investigated for the photoinactivation of *S. aureus* and *E. coli*. The bacterial suspensions were incubated and irradiated by visible light at different time intervals. This dyad inactivated more than 99% of *S. aureus* at a concentration of 5 μM in 30 min at 37 °C (Fig. 17b). The activity (25%) against the Gram-negative *E. coli* was found to be lower but significant in the same conditions.

**Fig. 18:**
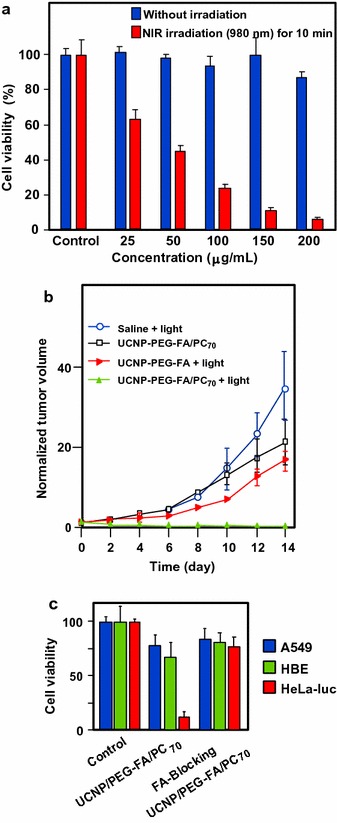
**a** Viability of HeLa-luc cells incubated with UCNP–PEG–FA/P–C_70_ at various concentrations. **b** Growth of tumors after treatments. The relative tumor volumes were normalized to their initial sizes. **c** Viability of A549, HBE, and HeLa-Luc cells incubated with either UCNP–PEG–FA/P–C_70_ or the mixture of UCNP–PEG–FA/P–C_70_ and an excess of folate (980 nm, 480 J/cm^2^) [Taken from reference (Guan et al. 2015a)]

Recently, Shu’s group (Guan et al. 2015a) included P–C_70_ dyads in upconversion NPs (UCNPs) which included a core of lanthanide (Gd, Y, Tm) wrapped electrostatically by P–C_70_ (P = trismethylpyridylporphyrin, TMPyP) and finally coated by folic acid-modified PEG. This UCNP–PEG–FA/P–C_70_ nanocomposite can act as a theranostic tool with folic acid (FA) as a targeting agent, PEG for furtivity, lanthanide for trimodal imaging (fluorescence/upconversion luminescence/magnetic resonance imaging), and P–C_70_ dyad for photoinduced therapy. The UV emission of UCNP matched with the absorption at 290, 345, and 361 nm, and visible emission at 451 and 475 nm of P–C_70_. The in vitro viability of HeLa-*luc* cells decreased with the concentration of UCNP–PEG–FA/P–C_70_ and 95% of cell death was obtained at a concentration of 0.2 mg/mL upon NIR irradiation (980 nm, 480 J/cm^2^) (Fig. 18a). Only 50% of cell death was observed in hypoxic condition. As illustrated in Fig. 18b, the in vivo growth of tumor cells was considerably decreased with UCNP–PEG–FA/P–C_70_ in the presence of light compared to controls.

**Fig. 19:**
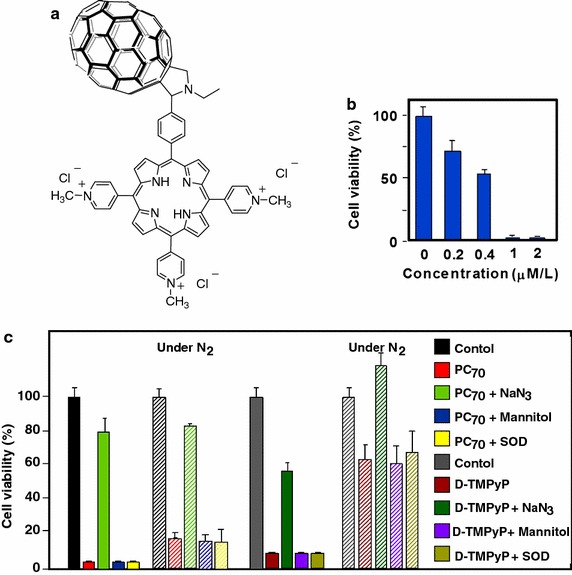
**a** Chemical structure of P–C_70_ dyad. **b** Viability of A549 cells incubated with P–C_70_ at gradient concentrations for 3 h and exposed to white light irradiation (17 mW/cm^2^, 10 min). **c** Viability of A549 cells incubated for 3 h with P–C_70_ and D-TMPyP (2 μM concentration) alone or in the presence of different ROS quenchers upon light irradiation for 10 min (white light irradiation, 17 mW/cm^2^) under air or nitrogen conditions [Taken from reference (Guan et al. 2015b)]

During the same year, Shu’s group (Guan et al. 2015b) revisited also the results on P–C_60_ dyad obtained by Alvarez and co-workers (2006) and synthesized an amphiphilic dyad porphyrin–C_70_ (P–C_70_) according to a previous methodology (Xu et al. 2011) by coupling the water-soluble 5-(4-formylphenyl)-10,15,20-tris(4-pyridyl)-porphyrin (D-TMPyP) with fullerene C_70_ via a Prato reaction (Maggini et al. 1993) (Fig. 19a). The P–C_70_ dyad formed a self-assembled liposomal structure with a diameter of ca. 30 nm. The P–C_70_ cellular uptake by A549 cells was threefold better than that of the porphyrin alone (D-TMPyP) and confocal microscopy showed that P–C_70_ was localized as small clusters in the cytoplasm. After 3-h incubation and 10-min irradiation at 405 nm (17 mW/cm^2^), the efficacy of cell killing was about 98% at a concentration of 1 μM/L under air atmosphere (Fig. 19b). More interesting is the efficiency of PDT under anaerobic conditions (Fig. 19c). Under the same conditions, but also nitrogen, the damages reached 80% for P–C_70_ and only 22% for D-TMPyP alone. Mechanism of this surprising PDT effect has been studied. While ^1^O_2_ is responsible for the damages in aerobic conditions, the formation of other ROS is involved under hypoxic condition, particularly from P–C_70_. The longer triplet lifetime of P–C_70_ (211.3 μs) can be assigned to the exciplex formed by energy transfer between the excited porphyrin and the ground state C_70_. All of these properties made P–C_70_ dyad an ideal candidate for anticancer PDT under shallow and hypoxic conditions. It has to be noted that Lee et al. (2001) published the synthesis of similar covalently linked chlorin–fullerene dyads formed by coupling methyl-pyropheophorbide-a and C_60_ in toluene at reflux without performing any PDT assays.

**Fig. 20:**
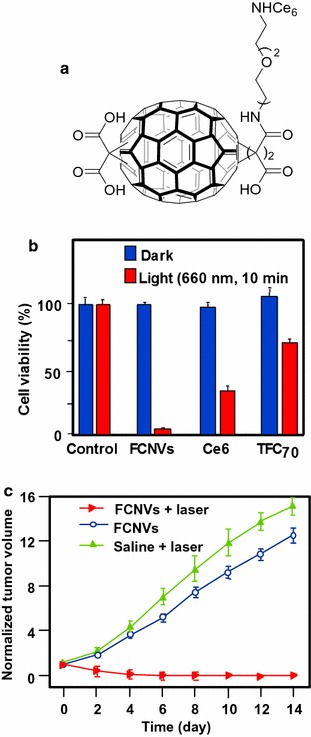
**a** Chemical structure of FCNVs. **b** Viability of A549 cells incubated with FCNVs, free Ce6, and TFC_70_ (0.1 mg/mL). **c** Growth of tumors after treatments. The relative tumor volumes were normalized to their initial sizes [Taken from reference (Guan et al. 2016)]

Very recently, the same group (Guan et al. 2016) described the production and the properties of fullerene C_70_ nanovesicles (noted FCNVs) made from C_70_–oligo ethylene glycol–Ce6 (Fig. 20a) which contained both hydrophilic and hydrophobic parts. These FCNVs have a high loading efficiency of Ce6 (57 wt% on tri-malonate derivative of fullerene C_70_ named TFC_70_) and efficient absorption in near-infrared spectroscopy could be observed. The diameter was estimated to be 31 nm as assessed by SEM, and AFM images indicated that the FCNVs are hollow spheres. In vitro experiments on A549 cells in the presence of NaN_3_ (^1^O_2_ quencher) clearly showed that cell death was due to ^1^O_2_. The ^1^O_2_ production was higher for the FCNVs than for Ce6 alone due to better absorption at 660 nm. Furthermore, negligible cytotoxicity was observed at concentrations up to 0.2 mg/mL. Figure 20b clearly shows the excellent ability of FCNVs to kill A549 cells as compared to Ce6 and TFC_70_ alone. Figure 20c shows the relative tumor volume 4 h after i.v. injection (10 mg/kg) and irradiation with 660-nm laser (0.1 W/cm^2^ for 10 min). The half-life of the FCNVs was shown to be 73.6 h (13.2 h for Ce6) indicating longer blood circulation than for the free Ce6.

**Fig. 21:**
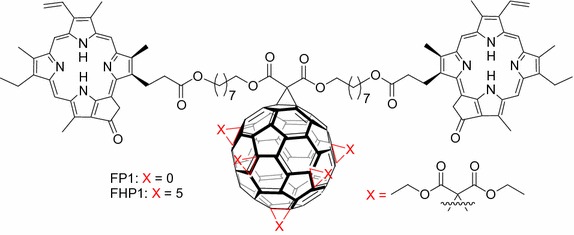
Chemical structures of mono- (FP1) and hexaadducts (FHP1) [Taken from reference (Rancan et al. 2005)]

Rancan et al. (2005, 2007a, b) proposed fullerene–pyropheophorbide-a conjugates as new tools for PDT. They synthesized different complexes starting from bis-malonato-pyropheophorbide-a up to decakis-pyropheophorbide-a[5:1]fullerene hexaadduct. They compared the uptake, photoinduced cytotoxicity, and photosensitizing activity of mono- (FP1) and hexaadducts (FHP1) towards human leukemia T lymphocytes (Jurkat cells) with free pyropheophorbide-a (Pyro-a) as a reference (Fig. 21) (Rancan et al. 2005).

Intracellular uptake of these derivatives showed a better accumulation than that of Pyro-a alone. This can be due to the size of adducts and a better diffusion through the plasma membranes of the small molecules when compared to the bigger ones, the uptake of which can occur only by endocytosis or pinocytosis. Nevertheless, the adduct FHP1 showed a better cytotoxic activity than FP1 with a 58% cell death when irradiated at 688 nm and 400 mJ/cm^2^.

**Fig. 22:**
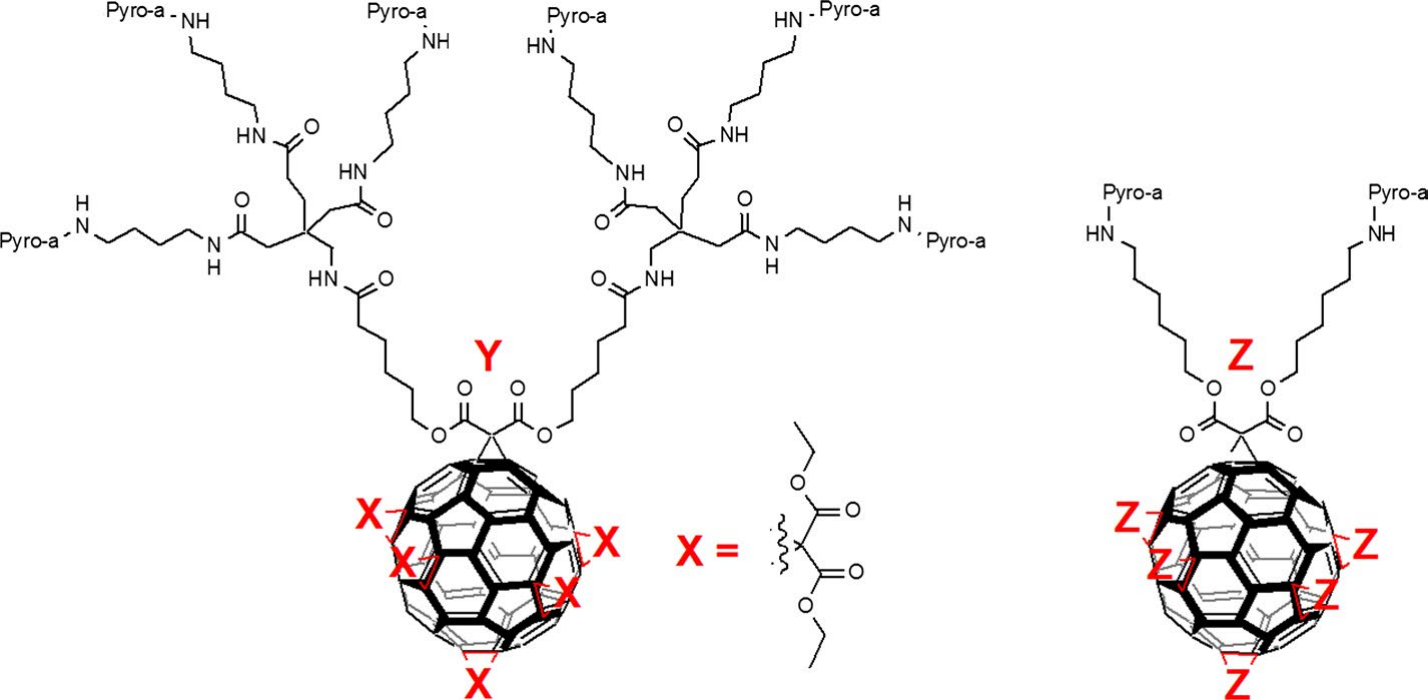
Chemical structures of hexamalonato-fullerenes with 6 Pyro-a units (FHP6) and 12 Pyro-a units (FHP12) [Taken from references (Rancan et al. 2007a, b)]

Two years later, the same group (Rancan et al. 2007a, b) described the synthesis of hexamalonato-fullerenes bearing 6 (FHP6) and 12 Pyro-a moieties (FHP12) (Fig. 22).

They showed that FHP6 exhibited a five times higher intracellular uptake than FHP1 and 40 times higher uptake than FHP12 but significantly lower than Pyro-a alone (Rancan et al. 2007b). In a last assay, they conjugated the decakis-pyropheophorbide-a[5:1]fullerene hexaadduct adipinic acid active ester with the monoclonal antibody Rituximab as an addressing unit. This antibody binds to the membrane of the CD20 receptor, which is overexpressed by cancer B cells. The affinity for the receptor was conserved as assessed by confocal microscopy (Rancan et al. 2007a). Unfortunately, the cell viability was 70% with Rituximab and the hexaadduct adipinic ester. No further results were published.

**Fig. 23:**
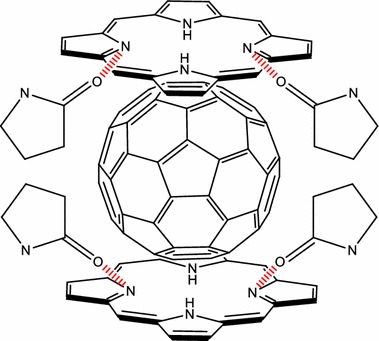
Chemical structure of TPP–PVP–C_60_ triad [Taken from reference (Ion et al. 2010)]

Ion et al. (2010) synthesized a tetraphenylporphyrin (TPP)–poly(vinylpyrrolidone) (PVP)–C_60_ (TPP–PVP–C_60_,) triad formulation (Fig. 23). The system was stabilized by electrostatic interactions between the three components, donor–acceptor bonds between C_60_, TPP, and PVP. In vitro studies have been performed on K562 leukemia cell lines: no dark toxicity was observed with a concentration up to 0.5 μM after 18 h of incubation. In the same conditions and under irradiation (436 nm, 0–1 J/cm^2^, 20–200 mW/cm^2^), only 20% of cells remained alive (80% without triad).

The in vivo experiments were performed on tumor-bearing rats (Walker 256 carcinoma) treated with TPP–PVP–C_60_ (10 mg/kg) (Ion et al. 2012). In tumor, the concentrations of lipid peroxides and protein carbonyls increased significantly, while those of the thiol groups decreased indicating a strong tumoral oxidative process. No further results were reported.

Guo et al. (2014) co-encapsulated malonic acid–fullerene (MA–C_60_) and docetaxel (DTX) in PEG–PLA micelles as delivery carriers. The average diameter was about 37 nm as assessed by TEM images. The viability of HeLa cells decreased with the co-entrapping of both MA–C_60_ and DTX (40% in the dark and 10% upon light irradiation at 339 nm after 72 h). After i.v. injection in S180 tumor-bearing mice, bioavailability of the MA–C_60_/DTX NPs was 2.25-fold higher than that of DTX micelles and a tumor growth inhibition rate of 81.3% at a 15 mg/kg dose was observed after 14 days (61.2% for DTX micelles).

**Fig. 24:**
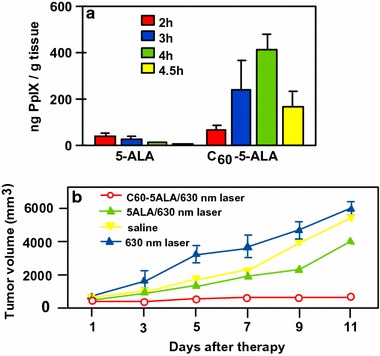
**a** PpIX synthesis from 5-ALA or C_60_–5-ALA in tissues as a function of time. **b** Growth of tumors after PDT treatment (630 nm, 0.1 W/cm^2^, 0.5 min) with 5-ALA or C_60_–5-ALA [Taken from reference (Li et al. 2014)]

Zhang’s group (Li et al. 2014) described 5-ALA-loaded fullerene vesicles (C_60_–5-ALA) obtained by dripping 5-ALA sodium salt into a toluene solution of fullerene C_60_. The diameter of the NPs has been estimated to be about 80–200 nm by DLS (Dynamic light scattering) and the loading of 5-ALA at the periphery of C_60_ was found to be 45 wt% as assessed by thermal gravimetric analysis. The PpIX generation was estimated after incubation, disruption, and extraction of B16-F10 cells (Fig. 24a). In tumor-bearing mice, PpIX induced by 5-ALA alone was found in almost all the tissues, while it is more selective for the tumoral tissue and lungs with C_60_–5-ALA. With 5-ALA alone, PpIX formation reached the maximum at 2 h and then rapidly decreased, while C_60_–5-ALA induced the maximum after 4 h. Furthermore, the authors observed an enhanced cell-killing effect with C_60_–5-ALA after irradiation at 630 nm (49% for C_60_–5-ALA and 32% for 5-ALA alone). Moreover, the tumor volume was stable after 11-day treatment, which was not the case for 5-ALA or irradiation alone (Fig. 24b).

Laptev et al. (2009) applied for a patent in which they described a pharmaceutical formulation for PDT of malignant tumors. They described the synthesis and the PDT assays of C_60_–PSs covalently bound to an amino acid or a dipeptide (Arg–Arg) and their combination with biocompatible synthetic biopolymers. Toxicity was only observed after 3–5 h post administration (adynamia, ruffling of hair, and absence of protecting reflex) but the animals return quickly to a normal state as assessed by histological analyses of the liver, kidney, spleen…. after 1 week of administration. The in vivo PDT bioassays (615–680 nm region) were carried out on BALB/c mice with lymphogenically metastasizing or hybrid mice F1 (CBA + C57/B6) with intraperitoneally transplanted embryocarcinoma. Qualitatively, the administration of the composition was characterized by the formation of a scab in the first 3–5 days and the necrosis in the tumor node which can reach 7–9 mm in depth.

### Fullerene: potential conjugates

**Fig. 25:**
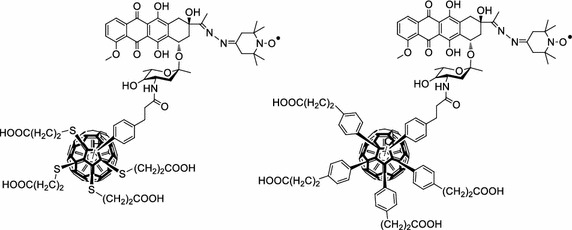
Conjugates between C_60_ and ruboxyl [Taken from reference (Kotelnikov et al. 2013)]

Kotelnikov et al. (2013) described two covalently linked conjugates between C_60_ and ruboxyl (Fig. 25). While ruboxyl and fullerene alone exhibit no or low PDT activity when irradiated at 500 nm, ruboxyl can interact with the fullerene moiety via energy and/or electron transfer. Thus, under visible irradiation at 500 nm, the resulting excited fullerene produced superoxide radical anion (O_2_^·‒^). Furthermore, the presence of a chlorine atom on the fullerene induced a 30% gain of the O_2_^·‒^ production as assessed by NBT (nitroblue tetrazolium) test. These potential PDT properties could be of interest for biomedical applications in cancer, virus, or bacterial treatments.

**Fig. 26:**
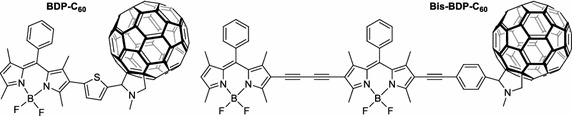
Light-harvesting fullerene dyads BDP–C_60_ and Bis-BDP–C_60_ [Taken from reference (Wu et al. 2012)]

As a promising tool in PDT and triplet–triplet annihilation upconversion (TTA-UC), Zhao’s team (Wu et al. 2012) described the photophysical behavior of two light-harvesting fullerene dyads as triplet PS (Fig. 26). Two conjugates BDP–C_60_ and Bis-BDP–C_60_ were synthesized from boron-dipyrromethene (BDP) and C_60_ by a Prato reaction. These heavy atom free molecules are efficient and exhibited upconversion quantum yields up to 7% and a strong absorption of visible light at 515 nm for BDP–C_60_ dyad and 590 nm for Bis-BDP–C_60_ dyad.

In the PDT field, the measurement of oxygen concentration during PDT is a particularly challenging problem due to real-time changes in oxygen demand and supply during the treatment. Starting from similar P–C_60_ dyad, Mermut et al. (2009) studied its use as a novel optical oxygen sensor in PDT. They related the measurement of O_2_ concentration during PDT by analyzing the magnetic field effect which can discriminate between the type I and type II photodynamic pathways, and proposed a new tool for controlling the intersystem crossing between singlet and triplet states using a fullerene linked to Zn or Cu porphyrin in liposome cell phantom.

Since their discovery in the 1980s, fullerenes (C_60_ and C_70_) have fascinated researchers. Fullerenes are semiconductor materials and used as organic photovoltaics, catalysts, antioxidants, in water purification, biohazard protection… and, quite recently, as nanomedicines. The formulation of this kind of nanomaterials involving fullerenes can be done in several ways, e.g., liposomes, micelles, dendrimers, or nanovesicles, and can be used in various fields of medicine such as antibacterial, PDT, PTT, or dual PDT/PTT agents. For example, functionalized fullerenes with carboxylic acid groups have found application in PDT as PSs due to their electron and energy transfer ability. Fullerenes can also act as *n*-type semiconductors (electron acceptors) and be conjugated with *p*-type porphyrins (electron donors) to develop dyads giving rise to a PDT effect mainly due to a light-induced electron transfer. At present, some researchers have started to develop bimodal protocols involving diagnosis/therapy, PDT/PTT, or aerobic/hypoxic conditions and these bimodal approaches seem to be the most promising. Table 4 below summarized the data available on the application of fullerene NPs grafted with or encapsulating PSs in PDT.

**Table 4:** Application of fullerene NPs in PDT

Type of NPs (size, nm)	PS (amount)	NPs–PS interactions	Irradiation conditions	Type of ROS	Cancer cell line		Refs.
					In vitro	In vivo	
Liposomes (nd)	ATMP (1/C_60_)	Conjugated	150 W lamp 350–800 nm, 54 J/cm^2^	^1^O_2_	Hep-2	‒	Alvarez et al. (2006)
Liposomes (nd)	AcTMP (1/C_60_)	Conjugated	150 W lamp 350–800 nm, 54 J/cm^2^	^1^O_2_	Hep-2	‒	Alvarez et al. (2009)
Reversed micelles (nd)	FTEP (1/C_60_)	Conjugated via 1,3-dipolar cycloaddition	Visible light (350–800 nm), 0.2 W/cm^2^, 8 J/cm^2^	^1^O_2_	*S. aureus* *E. coli*	‒	Belen Ballatore et al. (2014)
Liposomes (30)	D-TMPyP (1/C_70_)	Conjugated	White light 400–700 nm, 17 mW/cm^2^, 5–20 min	ROS ^1^O_2_	A549 HaCaT	‒	Guan et al. (2015b)
Liposomes (20)	TMPyP (1/C_70_)	Conjugated	Laser 980 nm, 0.8 W/cm^2^, 10 min	^1^O_2_	HeLa-*luc* A549 HBE	HeLa-*luc* tumor-bearing female BALB/c nude mice	Guan et al. (2015a)
Nanovesicles (31)	Ce6 (57 wt%)	Conjugated to C_70_ via the OEG2 linker	Laser 660 nm, 20 mW/cm^2^ (in vitro) or 0.1 W/cm^2^ (in vivo), 10 min	^1^O_2_	A549	4T1-*luc* tumor-bearing female BALB/c mice	Guan et al. (2016)
Micelles (37)	DTX (< 80 wt%)	Encapsulated	Diode laser 532 nm, 0.1 W/cm^2^, 5 min	ROS	HeLa	S180 tumor-bearing mice	Guo et al. (2014)
Supramolecular assemblies (nd)	TPP (nd)	H-bonds Electrostatic	Hg lamp, equipped with an UV39 filter, 436 nm, 20–200 mW/cm^2^, 0–1 J/cm^2^	nd	K562	‒	Ion et al. (2010)
Supramolecular assemblies (nd)	TPP (2/C_60_)	H-bonds Electrostatic	Red light 635 nm, 50 J/cm^2^, 15 min	ROS	‒	Walker 256 tumor-bearing Wistar rats	Ion et al. (2012)
Complexes (nd)	Ce6 and ZnCe6 (nd)	nd	A) Laser 662 nm and 890 nm, 100 J/cm^2^ B) Laser 615–680 nm	^1^O_2_	‒	A) Embryocarcinoma bearing F1 hybrid female mice (CBA and C57/B6) B) Lymphogenically metastasizing embryocarcinoma bearing female BALB/c mice	Laptev et al. (2009)
Complexes (nd)	Pyro-a (2/C_60_)	Conjugated	Laser diode 668 nm, 2.12 mW/cm^2^, 0.5 and 3 min	^1^O_2_	Jurkat	‒	Rancan et al. (2005)
Dendrimers (nd)	Pyro-a (6 or 12/C_60_)	Conjugated	nd	^1^O_2_	Jurkat Ramos EBV transformed B-lymphocytes	‒	Rancan et al. (2007a)
Dendrimers (nd)	Pyro-a (2, 6 or 12/C_60_)	Conjugated	Laser diode 668 nm, 2.12 mW/cm^2^, 0.5, 1 and 3 min	^1^O_2_	Jurkat	‒	Rancan et al. (2007b)
NPs (80–200)	5-ALA (45.12 wt%)	5-ALA complexed with C_60_	Laser 630 nm, 0.1 W/cm^2^, 0.5 min	nd	B16-F10	B16-F10 mice melanoma	Li et al. (2014)

*NPs* nanoparticles, *PS* photosensitizer, *ROS* reactive oxygen species, *nd* not disclosed, *ATMP* 5-(4-amidophenyl)-10,15,20-tris(4-methoxyphenyl)porphyrin, *AcTMP* 5-(4-acetamidophenyl)-10,15,20-tris(4-methoxyphenyl)porphyrin, *FTEP* 5-(4-formylphenyl)-10,15,20-tris[3-(*N*-ethylcarbozoyl)] porphyrin, *TMPyP* trismethylpyridylporphyrin, *D*-*TMPyP* 5-(4-formylphenyl)-10,15,20-tris(4-pyridyl)-porphyrin, *Ce6* chlorin e6, *OEG2* 1,10-diamino-4,7-dioxadecane, *DTX* docetaxel, *TPP* tetraphenylporphyrin, *UV* ultraviolet, *Pyro*-*a* pyropheophorbide a, *5*-*ALA* 5-aminolevulinic acid

## Graphene and graphene oxide

**Fig. 27:**
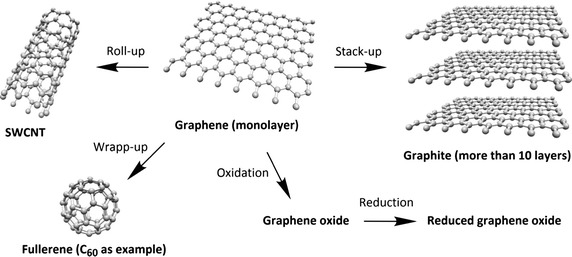
Chemical structures of graphene and its descendants

Graphene is a relatively new material recently isolated in 2004 by Novoselov et al. (2004), which has an extremely huge potential. It is a single layer of graphite, and its structure and physico-chemical properties permit its applications in different areas ranging from flexible electronics to DNA sequencing (Ahn and Hong 2014; Ojha et al. 2014; Raccichini et al. 2015). Starting from graphene, there are different two- and three-dimensional structures that can be produced and used as drug carriers (Fig. 27). Among these structures, we can cite single-walled carbon nanotubes (SWCNTs, rolled-up graphene monolayers), fullerenes (wrapped-up graphene, see fullerene NPs part), and graphite (stacked-up graphene monolayers). Graphene oxide (GO) and reduced graphene oxide (rGO) are, meanwhile, obtained after oxidation and oxidation/reduction processes, respectively (Fig. 27). Graphene is known, among other things, for its good electrical conductivity thanks to a two-dimensional (2D) network of sp^2^-hybridized carbon atoms.

Oxidation of graphene to GO causes the loss of the sp^2^ carbon network, i.e., GO becomes an electrical insulator, and the formation of several oxygen-containing functional groups (hydroxyl, carboxyl, and epoxy groups). The presence of oxygen atoms gives the GO better hydrophilicity than graphene, making it easier to disperse in organic solvents, water, and different matrixes. The reduction of GO to rGO is needed to restore electrical conductivity but this causes a considerable reduction of its dispersity due to its tendency to produce aggregates.

Numerous reviews report on the use of graphene or GO as drug carriers (Krishna et al. 2013; Dong et al. 2014; Wu et al. 2015). The first advantage relies on their large surface area. Another advantage arises from the *π*–*π* structure of graphene which can easily bind to aromatic structures, which is often the case for PS. In the field of cancer therapy and/or diagnosis, 73% of the articles focus on drug-delivering applications and 27% on theranostic applications (Orecchioni et al. 2015). In cancer therapy and particularly in PDT, some reviews (Shen et al. 2012; Goncalves et al. 2013; Rahman et al. 2015; Li et al. 2015a) can be useful for scientists.

### Graphene and single-walled carbon nanotubes (SWCNTs)

#### Graphene for PDT applications

Liu et al. (2015a) published an interesting article describing the direct one-pot synthesis of graphene (G) loaded with Ce6 via *π*–*π* stacking interactions by a simple sonification of Ce6 and graphite in an aqueous solution (loading efficiency 160 wt% and exfoliation yield 9%). During the Ce6 loading, graphite is progressively exfoliated to G to form G–Ce6 nanocomposite. In addition to having the advantage of directly producing graphene-based PS without going through an oxidizing step to generate GO, the authors found that the G–Ce6 displays remarkable characteristics. First, the Ce6 loading for G is tenfold higher than that for GO analogues, and no functionalization of G is required to obtain good dispersibility in physiological conditions. The in vitro study of G–Ce6 (HeLa cells, laser irradiation 660 nm for 2 min) provided evidence of ROS generation and showed that the concentration necessary to kill cells with G–Ce6 is 6–75 times lower than in any other Ce6 composites including GO–Ce6. This result opens the path to drawing up new graphene-based PS as nanocarriers for PDT.

Wu et al. (2014) studied the synergistic activity of polylysine–graphene (G–PLL) formulated with doxorubicin (Dox) and Zn(II) phthalocyanine (ZnPc). The nanocomplex was easily prepared by self-assembling ZnPc and Dox on G–PLL and showed high solubility and stability in biological media. It was found that the ^1^O_2_ production was lower than that of ZnPc alone, but besides it was also shown that the activity of the PS may be restored after release (Zhu et al. 2008; Tian et al. 2011). The cytotoxicity to HeLa cells was found to be negligible; however, when subjected to irradiation (660 nm, 0.15 W/cm^2^ for 10 min), a 90% loss of viability was observed with an IC_50_ of 0.14 μg/mL. This synergistic effect was also observed with MCF-7 (IC_50_ = 0.21 μg/mL) and B16 mouse melanoma cells (IC_50_ = 0.28 μg/mL).

#### SWCNTs and PDT applications

**Fig. 28:**
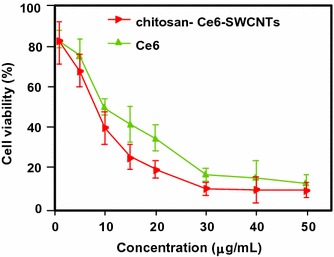
In vitro PDT effect of SWCNTs–Ce6–chitosan on HeLa cells (663 nm, 150 mW/cm^2^, 10 min) [Taken from reference (Xiao et al. 2012)]

Zhu’s team (Xiao et al. 2012) studied the chemical characteristics and PDT efficacy of SWCNTs–Ce6–chitosan nanorods. The obtained nanorods had an overall diameter of 6–7 nm with a thickness of PS of about 1.2 nm. The dark toxicity of SWCNTs–Ce6–chitosan (100 μg/mL) was found to be less than 20% after 48-h incubation with NIH/3T3 normal cells. The PDT effect of SWCNTs–Ce6–chitosan to HeLa cells was determined in vitro by WST-1 assay and showed that the IC_50_ value of free Ce6 is about 8.80 ± 0.059 μg/mL, while that of the nanocomposite is only 5.98 ± 0.064 μg/mL probably due to better hydrophilicity (Fig. 28).

**Fig. 29:**
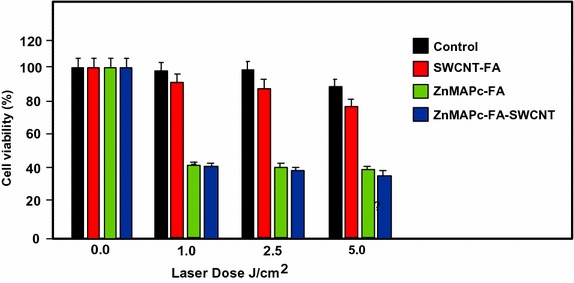
Viability of melanoma cells incubated with SWCNT–FA, ZnMAPc–FA, and ZnMAPc–FA–SWCNT under irradiation (676 nm, 98 mW/cm^2^, 5 J/cm^2^) [Taken from reference (Ogbodu et al. 2015b)]

Ogbodu and co-workers (2014; Ogbodu et al. 2013a, b, 2015a, b) studied SWCNTs with 1–5 nm diameter and 1–5 μm length as nanocarriers of several zinc phthalocyanine–X (ZnPc–X) conjugates (Table 5). These conjugates were adsorbed onto SWCNT via *π*–*π* stacking interactions. They presented better photophysical properties than the ZnPc alone. The in vitro phototoxicity experiments were performed with ZnMAPc–FA–SWCNT and ZnMAPc–spermine–SWCNT on human skin melanoma A375 and MCF-7 breast cancer cells, respectively. After diode laser irradiation (676 nm, 98 mW/cm^2^, 5 J/cm^2^) in the presence of ZnMAPc–FA–SWCNT at 10 μM, a PDT effect was observed with a 37% cell viability compared to 40% with ZnMAPc–FA and 77% with SWCNT–FA, showing that SWCNT does not have a significant PDT or PTT effect on the cells (Fig. 29).

**Table 5:** ZnPc–X conjugates

ZnPc	X	Refs.
ZnMAPc	Pyrene	Ogbodu et al. (2013b)
ZnMAPc	Folic acid	Ogbodu et al. (2013a, 2015b)
ZnOPc	Bovine serum albumin	(Ogbodu and Nyokong 2014)
ZnMCPPc	Spermine	(Ogbodu et al. 2015a)

*ZnMAPc* zinc monoamino phthalocyanine, *ZnOPc* zinc octacarboxy phthalocyanine, *ZnMCPPc* zinc monocarboxyphenoxy phthalocyanine

**Fig. 30:**
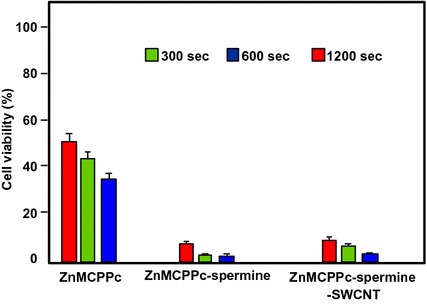
Viability of MCF-7 breast cancer cells incubated with ZnMCPPc, ZnMCPPc–spermine, and ZnMCPPc–spermine–SWCNT at 40 μM concentration under irradiation (600–1000 nm, 93 mW/cm^2^, 112 J/cm^2^) for 20 min [Taken from reference (Ogbodu et al. 2015a)]

Concerning ZnMAPc–spermine–SWCNT, the same authors decided to use a quartz lamp that can absorb sunlight from about 600 nm and beyond 1000 nm in order to investigate the PDT and PTT effects of ZnMAPc and SWCNT by varying different parameters, such as concentrations of ZnMCPPc, ZnMCPPc–spermine, or ZnMCPPc–spermine–SWCNT (from 5 to 40 μM) and irradiation time (5, 10, or 20 min) equivalent to an irradiation dose of 28–112 J/cm^2^. The best results were obtained at 40 μM concentration after 20-min irradiation. These results indicated that ZnMCPPc–spermine–SWCNT exhibited 5% cell viability but ZnMCPPc–spermine and ZnMCPPc showed 3% and 36%, respectively. ZnMCPPc–spermine has a better PDT effect compared to ZnMCPPc–spermine–SWCNT, as it possesses higher triplet and ^1^O_2_ quantum yield values, and no clear PTT effect of SWCNTs was observed (Fig. 30).

Also in respect of SWCNTs, Safar et al. (2015) wished to combine chirality-enriched (6,5) single-walled carbon nanotubes (E-SWCNTs) and some porphyrins for estimating the potential PDT effect of these new hybrid systems. To achieve this goal, *meso*-tetrakis(4-pyridyl)porphyrin tosylate salt (H_2_TM4PyP (OTs)_4_, POR) and its myristyl analogue *meso*-tetrakis(*N*-myristyl-4-pyridinium)porphyrin tosylate salt (H_2_TMy4PyP (OTs)_4_, MYR) were chosen. Commercial Verteporfin (VER) was used as the reference. After studying the optical absorption of porphyrins and E-SWCNT (Table 6), the production of ^1^O_2_ for each hybrid system was evaluated using a white 5-LED lamp with a yellow or red filter (wavelength = 570 and 630 nm, respectively) or 940 nm. The authors observed that E-SWCNT alone can produce ^1^O_2_, involving very likely a direct energy transfer from E-SWCNT excitons to dissolved oxygen, but less than the hybrid systems. Furthermore, it has been shown in some cases that the hybrid systems have a better ^1^O_2_ production efficiency that the free porphyrins in the therapeutic window.

**Table 6:** Optical absorption of porphyrins and E-SWCNT

Compounds^a^	Absorption bands (nm)^b^
POR	521, 556, 587, and 641
MYR	530 and 570
VER (commercial, reference)	575, 630, and 695
E-SWCNT	572 and 992^c^

*POR meso*-tetrakis(4-pyridyl)porphyrin tosylate salt, *MYR meso*-tetrakis(*N*-myristyl-4-pyridinium)porphyrin tosylate salt, *VER* verteporfin, *E*-*SWCNT* chirality-enriched (6,5) single-walled carbon nanotube

^a^Porphyrin aqueous solutions at 55 μM and aqueous suspension of E-SWCNT

^b^For all porphyrins, another weak band can be observed from 900 to 1000 nm

^c^Bands attributed to E22 and E11 optical transitions (Weisman and Bachilo 2003)

#### PDT/PTT applications

Some research teams worked on the use of photothermal effect of PS–graphene nanocomposites for the development of new theranostic nanoplatforms by combining PDT and PTT (Yang et al. 2010) to improve the efficacy against cancer.

**Fig. 31:**
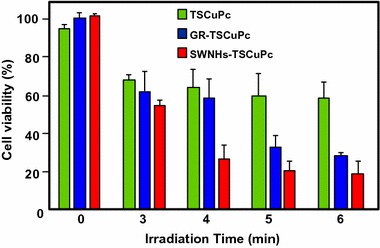
Viability of HeLa cells incubated with free TSCuPc, GR–TSCuPc, and SWNHs–TSCuPc nanohybrids (650 nm, 3 W/cm^2^) [Taken from references (Jiang et al. 2014a, b)]

Jiang and co-workers synthesized two new PS nanocarriers with a dual phototherapy effect (PDT/PTT) in one sonication step by coating tetrasulfonic acid tetrasodium salt copper phthalocyanine (TSCuPc) onto graphene sheets (GR) (2014b) and single-walled carbon nanohorns (SWNHs) (2014a) via *π*–*π* interactions. The loading efficiencies of TSCuPc were 27 and 38 wt% for GR–TSCuPc and SWNHs–TSCuPc nanohybrids, respectively. Both nanohybrid systems produce ROS, such as superoxide radical anion (O_2_^·‒^) and hydroxyl radical (OH·), and their in vitro dual phototherapeutic effects (PDT/PTT) at the TSCuPc equivalent of 10 μg/mL using human cervical cancer HeLa cells were highlighted using a single wavelength (laser irradiation at 650 nm, 3 W/cm^2^ for 5 min) (Fig. 31).

**Fig. 32:**
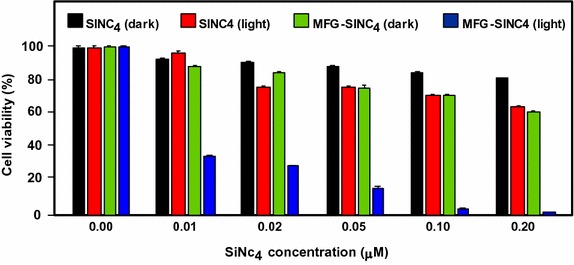
Comparative cell viabilities by MTT assay of HeLa cells treated with different concentrations of SiNc4/MFG–SiNc_4_ under dark/photoirradiation conditions (750–1380 nm, 0.3 W/cm^2^, 1 h) [Taken from reference (Gollavelli and Ling 2014)]

Gollavelli and Ling (2014) used a graphene derivative loaded with a PS by *π*–*π* stacking interactions in order to develop a new theranostic nanoplatform combining phototherapy (PDT and/or PTT) and imaging for cancer treatment and detection. Water-dispersible magnetic and fluorescent graphene NPs (MFG) and hydrophobic silicon napthalocyanine bis(trihexylsilyloxide) (SiNc_4_) as PS were chosen to achieve their goals (MFG size ≈ 40 nm, loading efficiency of PS 8.5 wt%). The Magnetic Resonance Imaging (MRI) measurements and the in vitro phototherapy study (PDT and PTT) were conducted on human cervical cancer HeLa cells (Fig. 32). The authors proved that MGF–SiNc_4_ was well internalized in HeLa, and T_2_-weighted MRI measurements revealed a great luminescence image and T_2_-weighted MRI contrast due to the fluorescence and superparamagnetic properties of MFG. It should be noted that MFG and SiNc_4_ can together absorb ≈ 775 nm light and it was demonstrated that a dual phototherapy effect (PDT and PTT) is possible to kill cells using an inexpensive single light source (tungsten halogen lamp equipped with a long pass filter capable of providing a light wavelength range of 750–1380 nm and delivers a power of 0.3 W/cm^2^ for 1 h). This experiment indicated a cancer cell-killing efficacy of ≈ 97.9% (PDT ≈ 64.7% and PTT ≈ 33.2%).

### Graphene oxide

#### PDT applications

Dong et al. (2010) described the use of methoxy-poly(ethylene glycol)-conjugated nano-graphene oxide (NGO–mPEG) as a potential PS nanocarrier for anticancer PDT. Hydrophilic mPEG was conjugated to the NGO for increasing the solubility and stability of NGO in cell culture media. The authors found that the structure of NGO appeared like single-layer sheets with size down to 200 nm and thickness of about 2–3 nm and the cytotoxicity of NGO–mPEG towards human breast cancer MCF-7 cells was negligible. ZnPc as PS was loaded onto NGO–mPEG by *π*–*π* stacking and hydrophobic interactions (loading efficiency 14 wt%) to evaluate the in vitro photodynamic effect of NGO–mPEG/ZnPc. Human MCF-7 breast cancer cells were treated by different concentrations of NGO–mPEG/ZnPc for 24 h followed by an exposition to the UV band-path filtered Xe light irradiation for 10 min (60 J/cm^2^). Without light irradiation, the authors observed a cell viability of > 85% and, with irradiation, this value decreases from about 80 to 60% when the concentration was increased from 3.8 to 60 mg/L.

Wojtoniszak et al. (2013) showed that GO loaded with methylene blue (MB) as a PS by adsorption had a much higher ^1^O_2_ generation capacity than graphene after irradiation with 785-nm laser. They concluded that MB–GO could be used as a potential PS delivery system in PDT. No in vitro and in vivo studies have been conducted yet.

Miao et al. (2013) demonstrate that polyethylene glycol-grafted graphene oxide (pGO) nanosheets can be employed as the multimodal nanocarrier to co-deliver a PS Ce6 and Dox to induce synergystic photodynamic anticancer effect. The PEGylation of pGO is necessary to increase aqueous stability of pGO. Ce6 and Dox were co-loaded onto pGO nanosheets by *π*–*π* stacking and hydrophobic interactions (loading efficiencies 51.9 ± 5.1 and 61.7 ± 4.4 wt%, respectively) to form multimodal nanophysisorplexes (Ce6/Dox/pGO) with a size of 148.0 ± 18.0 nm. The in vitro cytotoxicity of GO and pGO nanosheets towards murine SCC7 squamous carcinoma cells was evaluated after treatment with a concentration of 40 μg/mL and the cell viability is around 90% for both. Conversely, the in vivo cytotoxicity, after treating mice by intravenous bolus injection of nanosheets at 80 mg/kg dose, showed no cytotoxicity for pGO nanosheets compared to the 10% survival rate for GO nanosheets. The authors proved by in vivo and in vitro studies (irradiation with 660-nm LED with a luminous intensity of 8000 mCd for 30 min) that the co-delivery of Ce6 and Dox by the pGO nanosheets induces a synergistic photodynamic anticancer effect and the best result was obtained at a Dox–Ce6 molar ratio of 1:2.

**Fig. 33:**
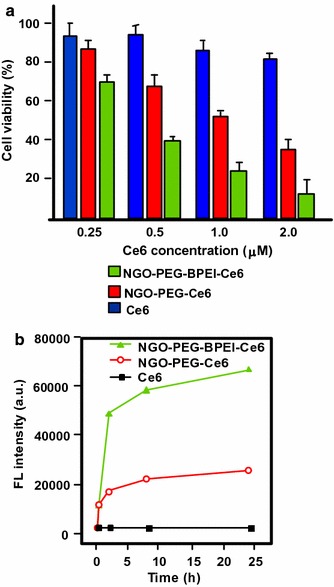
**a** Viability of HeLa cells treated with free Ce6, NGO–PEG–Ce6, and NGO–PEG–BPEI–Ce6 at different concentrations of Ce6. **b** Ce6 fluorescence intensity in HeLa cells treated with Ce6, NGO–PEG–Ce6, and NGO–PEG–BPEI–Ce6 for various times [Taken from reference (Miao et al. 2013)]

As did Dong and co-workers (2010), Zeng et al. (2015) also synthesized PEGylated nano-graphene oxide (NGO–PEG). The PEG-amine was covalently attached to NGO sheets for the dispersibility improvement of NGO in physiological conditions. The size of NGO–PEG is around 20–40 nm with a thickness ≈ 1.4 nm. The loading of Ce6 onto NGO–PEG by *π*–*π* stacking and hydrophobic interactions (loading efficiency ≈ 13 wt% at the feeding concentration of 2 mM) and in vitro PDT study (HeLa cells, laser irradiation 660 nm, 0.2 W/cm^2^ for 5 min) showed that NGO–PEG can be used as a PS nanocarrier. Furthermore, when coupled to branched polyethylenimine (BPEI) the resulting positively charged NGO–PEG–BPEI showed a better cellular uptake by HeLa cells and the loading ratio of Ce6 reached a value of 26 wt%. For the cellular uptake by HeLa cells, cytometric analysis, confocal microscopy, and fluorescence experiments were carried out. Figure 33a shows the viability of HeLa cells after light irradiation by 662-nm laser at a power density of 0.2 W/cm^2^ for 5 min and Fig. 33b shows the changes of Ce6 fluorescence intensity in cells as a function of incubation time. It can be noted that the mean fluorescence intensity of Ce6 in the NGO–PEG–BPEI–Ce6 system was 2.6-fold higher than that in NGO–PEG–Ce6 and 30-fold better than that in the free Ce6 system. This preliminary study indicated that NGO–PEG–BPEI–Ce6 could target the delivery of Ce6 into lysosome.

**Fig. 34:**
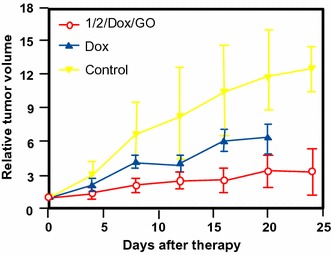
Tumor growth curves for BALB/c nude mice with HeLa cancer cells [Taken from reference (Yang et al. 2012)]

Yang et al. (2012) described a scaffold for PDT and drug delivery in which GO is the carrier, folic acid-modified cyclodextrin (FA-βCD) is a targeting agent, Dox is the drug, and adamantanyl porphyrin (AdaTPP) is a linker. The assembly of the nanocomposite resulted from successive mixings of GO, AdaTPP, and Dox to form GO–Dox/AdaTPP, then with FA-βCD by strong hydrophobic interaction between the CD cavity and adamantane moiety. The resulting assembly 1/2/Dox/GO showed no cytotoxicity towards normal cells over a 1-day period but induced tumor growth inhibition in vivo in HeLa-bearing BALB/c nude mice as illustrated in Fig. 34.

**Fig. 35:**
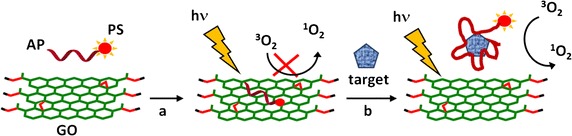
Concept of photomolecular beacon [Taken from reference (Yan et al. 2014)]

Yan et al. (2014) reported the regulation of ^1^O_2_ production probe using the binding properties of an aptamer (AP). The concept of this molecular beacon [for a review, see (Verhille et al. 2010)] is based on a non-covalent binding of a cDNA aptamer–Ce6 (AP–Ce6) moiety with GO as an efficient fluorescence and SOG (singlet oxygen generation) quenching probe. In the absence of a target, the PS is not active due its close proximity to GO. Once the AP hybridizes with the target, the ^1^O_2_ production is restored (Fig. 35).

**Fig. 36:**
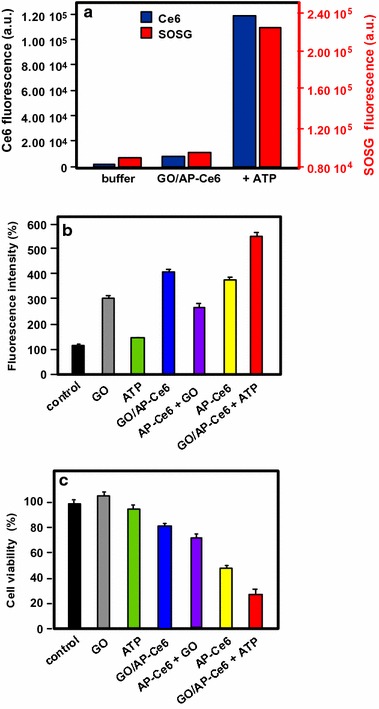
**a** The Ce6 and SOSG signal readouts after irradiation condition (404 nm, 10 min). **b** DCF fluorescence intensity in HepG2 cells. **c** Cellular viability of HepG2 cells treated with light [Taken from reference (Yan et al. 2014)]

Adenosine-5′-triphosphate (ATP), which is overexpressed on the extracellular surface of cancer cells besides the mitochondrial matrix, was used as a proof-of concept target molecule. The graphene-based GO/AP–Ce6 was prepared by mixing GO and AP–Ce6 and exhibited a size of 80 nm and a thickness of about 1.2 nm. The quenching by graphene is clearly evidenced in Fig. 36a, and it can be noted that the presence of ATP as a target restored the ability of Ce6 to produce ^1^O_2_ owing to stronger interactions between ATP and AP–Ce6 than those of GO and the PS. In human HepG2 cells, ^1^O_2_ was detected using DCFH-DA (2′,7′-dichlorodihydrofluorescein diacetate) method and the results evidenced the restoration of ^1^O_2_ production in the presence of ATP (Fig. 36b). These results are comforted by cell viability after light irradiation at 404 nm for 10 min (Fig. 36c).

A targeted ligand is often required to provide a better selectivity of the PS. Within this scope, some teams developed targeted nanocarrier systems for the PS delivery.

Huang and co-workers (2011) prepared GO as single-layer sheet (sheet-like shapes) with a thickness of about 1.2 nm covalently linked to folic acid (FA). The PS (Ce6) was then loaded onto FA–GO (loading efficiency ≈ 80 wt%) via hydrophobic interactions and *π*–*π* stacking. The in vitro cytotoxicity of FA–GO and FA–GO–Ce6 was measured after 24-h incubation with MGC803 cells and showed that GO–FA does not have any dark toxicity and the dark cytotoxicity of FA–GO–Ce6 is dependent upon Ce6 concentration. The intracellular distribution study revealed a higher accumulation of Ce6 inside the tumor. The in vitro PDT effect was studied after the exposure of MGC803 cells to FA–GO–Ce6 at different ratios (FA–GO:Ce6, 10:1 to 1:1 wt%) followed by irradiation with a He–Ne laser (632.8 nm, ≈ 30 mW/cm^2^) for 10 min. Without irradiation, cell viability above 80% was observed and after light exposure only ≈ 10% of cell viability was detected for 1:1 and 2:1 ratios.

**Fig. 37:**
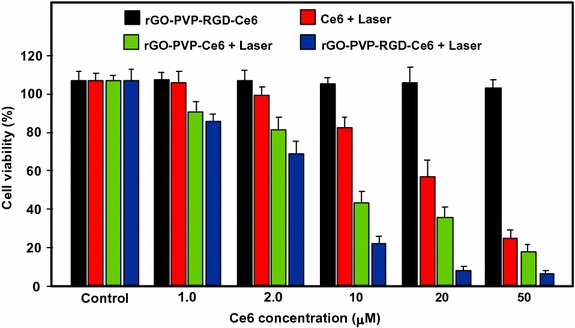
Viability of MGC803 cells treated with different concentrations of Ce6, rGO–PVP–Ce6 or rGO–PVP–RGD–Ce6 for 24 h at 37 °C without or with irradiation (671 nm, 30 mW/cm^2^) [Taken from reference (Huang et al. 2015)]

The same team (Huang et al. 2015) described the preparation of reduced graphene oxide (rGO) nanosheets covalently linked to polyvinylpyrrolidone (PVP). PVP was used for increasing aqueous dispersibility and biocompatibility of rGO. The rGO–PVP shows a sheet-like shape with PVP layers of thickness ≈ 0.85 nm on each side of the 0.96 ± 0.05-nm-thick rGO layers (total thickness for rGO–PVP 2.81 ± 0.18 nm). The ACDCRGDCFCG peptide (RGD4C), used as vectors for α_v_β_3_ integrin-targeted delivery of PSs, was covalently anchored to rGO–PVP and the resulting rGO–PVP–RGD was loaded with Ce6 by hydrophobic interactions and *π*–*π* stacking (loading efficiency ≈ 80 wt%). No dark cytotoxicity of rGO–PVP and rGO–PVP–RGD on MGC803 cells was observed in the concentration range of 0–500 μg/mL (cell viability higher than 95%). The authors also observed that rGO–PVP–RGD–Ce6 induced a better PDT effect on cells as compared to free Ce6 and rGO–PVP–Ce6 at all tested concentrations (0–50 M Ce6, i.e., 0.6–30 μg/mL Ce6) after laser irradiation (671 nm, 30 mW/cm^2^ for 3 min) (Fig. 37). These results demonstrated the active targeting ability of RGD. The ^1^O_2_ generation from rGO–PVP–RGD–Ce6 has been detected by measuring the fluorescence signal of singlet oxygen sensor green (SOSG).

Li et al. (2013) covalently attached hyaluronic acid (HA) onto the surface of GO nanosheets in order to target cancer cells with overexpressed HA receptors. The hydrodynamic diameter of GO nanosheets is 59.3 nm and the average diameter of HA–GO nanocarrier is 78.1 nm. Ce6 was loaded onto HA–GO via *π*–*π* stacking and/or hydrophobic interactions (loading efficiency 115 wt% at the feeding concentration of 1.5 mg/mL). The in vitro phototoxicity study (laser irradiation 670 nm, 1.8 J/cm^2^) of HA–GO/Ce6 nanohybrids towards human cervical cancer HeLa cells showed a photodynamic efficacy ten times greater than free Ce6 (IC_50_ shifted from 1 to 0.1 μg/mL).

**Fig. 38:**
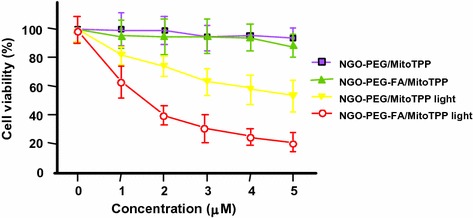
Viability of HeLa cells after treatment with NGO–PEG/MitoTPP in the presence/absence of FA and light (650 nm, 10 mW/cm^2^, 30 min) [Taken from reference (Xu et al. 2015)]

Xu et al. (2015) described a dual-targeting nanosystem comprising a modified nano-graphene oxide (NGO) as the carrier and a PS (5-(*p*-(4-trimethylammonium)-butoxyphenyl)-10,15,20-triphenylporphyrin bromide abbreviated MitoTPP). The NGO nanocomposite was modified as follows: Firstly, 4-arm PEG-amine was covalently coupled as described by Li et al. (2013) to enhance dispersibility and biocompatibility, and secondly folic acid (FA) NHS ester was grafted on NGO–PEG to give the modified NGO–PEG–FA composite. Finally, an overnight mixing of NGO–PEG–FA and MitoTPP afforded the desired nanosystem NGO–PEG–FA/MitoTPP (loading of MitoTPP was estimated to be 37.2 wt%). The in vitro release of MitoTPP was strongly dependent on pH. Due to the non-ionized carboxyl groups in NGO at pH 5.0, the electrostatic attraction is much weaker and promotes the release of MitoTPP from NGO and permits to escape from the acidic endosome/lysosome compartments and then diffuse into mitochondria. Confocal microscopy showed that the intracellular localization of MitoTPP was found to be mitochondria. ^1^O_2_ generation was observed using ABDA (9,10-anthracenediyl-bis(methylene)dimalonic acid) indicator at pH 7.4 and 5.0 under irradiation and over different periods. While the absorbance of ABDA did not change at pH 7.4 for 1 h, the one at pH 5.0 dramatically decreased within the initial 15-min irradiation, indicating that MitoTPP can induce ^1^O_2_ after being released from NGO. The presence of FA in the nanocomposite induced a much brighter fluorescence than with NGO–PEG/MitoTPP demonstrating dual targeting. The phototoxicity caused by PDT is presented in Fig. 38 which clearly shows the greater effectiveness of this double targeting after 30 min of light irradiation (650 nm, 10 mW/cm^2^).

Zhou and colleagues (2015) worked on the development of a photosensitive and magnetically targeted PS delivery system by dispersing in a uniform size the Fe_3_O_4_ magnetic NPs on the GO surface by co-precipitation method. The thickness of the single-layer sheet was about 0.8 nm and the saturation magnetization value of the resulting GO–Fe_3_O_4_ nanocomposite was about 15 emu/g, preventing any Fe_3_O_4_ aggregation on GO. Hematoporphyrin (HP) was loaded onto GO–Fe_3_O_4_ by hydrophobic interactions and *π*–*π* stacking (loading efficiency 23.6 wt%). The authors determined that GO–Fe_3_O_4_ produces ^1^O_2_ but in smaller quantities compared to HP alone (63.4% of the value obtained with free HP), which is in agreement with the literature (Tian et al. 2011). The in vitro study on human cervical cancer HeLa cells highlighted the fact that the IC_50_ value of GO–Fe_3_O_4_–HP decreased from 189.24 μg/mL (dark cytotoxicity) to 10.12 μg/mL after incubating the cells with GO–Fe_3_O_4_–HP for 24 h followed by laser irradiation at 671 nm (0.1 W/cm^2^) for 5 min and continued incubation for 72 h. These results clearly demonstrate that the magnetic GO–Fe_3_O_4_ nanocomposite could be used as the tumor-targeted PS delivery system under proper external magnetic field and as a photosensitive PDT agent under laser irradiation at 671 nm to produce ^1^O_2_.

**Fig. 39:**
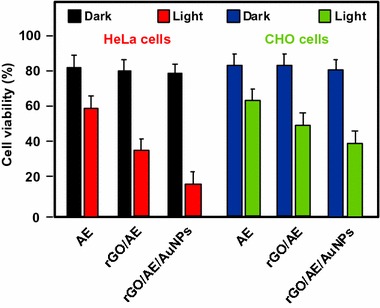
Viability of HeLa and CHO cells treated with AE, rGO/AE, and rGO/AE/AuNPs in the dark or after light irradiation (600 nm, 2 W/cm^2^ for 10 min) [Taken from reference (Chang et al. 2015)]

Recently, Chang et al. (2015) described a new concept using near-infrared (NIR) irradiation as a stimulus for the formation of the hydrogel shells inducing cell death. The developed hydrogel precursor (rGO/AE/AuNPs) was composed of reduced graphene oxide (rGO), amaranth extract (AE), and gold nanoparticles (AuNPs). AuNPs and rGO were used for their PTT properties and ability to speed up the ^1^O_2_ generation. AE was used as a PS (due to the chlorophyll derivatives contained in the extract) and as a cross-linking agent. The authors showed that the hydrogel precursor was initially in the form of a viscous oil, but a rapid hydrogel formation was observed upon 10-min NIR irradiation. This hydrogel formation can be ascribed to several interactions between the various constituents of the hydrogel precursor, i.e., excess of AE inducing reduction of GO to rGO, formation of dewatered rGO sheets caused by an increase in the temperature of precursor under NIR irradiation, improvement of the *π*–*π* stacking and hydrophobic interactions among rGO sheets, and finally electrostatic attraction between positively charged Au NPs and negatively charged GO sheets. In vitro studies on Chinese hamster ovary (CHO) and HeLa cancerous cells showed an in situ hydrogel shell formation on cells after laser irradiation (600 nm, 2 W/cm^2^ for 10 min), which led to an enrichment of PS and PTT agents around the tumor cells resulting in cell death via a dual PDT/PTT treatment with minimal side effects (Fig. 39).

**Fig. 40:**
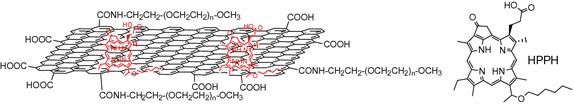
Schematic structure of the GO–PEG–HHPH system [Taken from reference (Rong et al. 2014)]

The same year, Rong and co-workers (2014) used polyethylene glycol-functionalized graphene oxide (PEG–GO) as a PS delivery system. PEG–GO was loaded with 2-(1-hexyloxyethyl)-2-devinyl pyropheophorbide-alpha (HPPH, Photochlor^®^) as a PS (loading efficiency 131 wt%) by supramolecular *π*–*π* stacking. The PEG–GO system was of about 50 nm and has a thickness of around 1.5 nm, which reaches up to 2 nm after HPPH loading (Fig. 40).

**Fig. 41:**
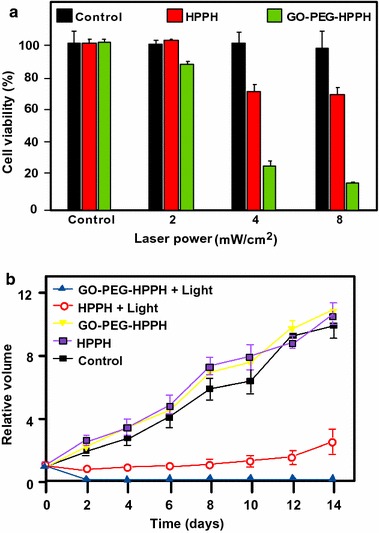
**a** Viability of 4T1 cells incubated with various concentrations of free HPPH, GO–PEG, and GO–PEG–HPPH after irradiation at 671 nm (4 mW/cm^2^, 3 min). **b** Tumor growth curves of different groups of tumor-bearing mice after PDT treatment. The tumor volumes were normalized to their initial values [Taken from reference (Rong et al. 2014)]

The in vitro PDT studies of GO–PEG–HPPH, GO–PEG, and HPPH alone towards murine breast cancer 4T1 cells were performed after incubation with GO–PEG–HPPH (1 μm equivalent of HPPH and 0.49 μg/mL of GO–PEG) for 24 h followed by laser irradiation at 671 nm (4 mW/cm^2^) for 3 min. No dark cytotoxicity was observed, and after irradiation GO–PEG–HPPH caused a greater cell death than HPPH alone (Fig. 41a). The in vivo HPPH delivery and PDT effects of GO–PEG–HPPH and free HPPH were studied in 4T1 tumor-bearing mice with both fluorescence and PET (positron emission tomography) imaging. While there was a reduction of the generation of ^1^O_2_ by GO–PEG–HPPH compared to the free HPPH, a better accumulation of HPPH into the tumor region was observed. The in vivo PDT study was carried out after an i.v. injection of GO–PEG–HPPH or free HPPH ([HPPH] = 1 mg/kg in both cases) followed by laser irradiation at 24 h post injection (671 nm, 75 mW/cm^2^ for 20 min). A marked improvement in average life expectancy has also been observed (35% after 60 days). Subsequently, the volume of tumors in the GO–PEG–HPPH-treated mice decreased in the first 2 days and remained unchanged for at least 14 days (Fig. 41b).

**Fig. 42:**
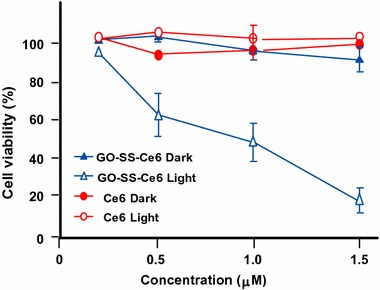
In vitro PDT efficacy on A549 cells of free Ce6 and GO–SS–Ce6 system under dark or light irradiation conditions (670 nm, 20 J/cm^2^) [Taken from reference (Cho and Choi 2012)]

Cho et al. (2012) linked GO to Ce6 with disulfide bond (SS) to generate GO–SS–Ce6 system having an average hydrodynamic size of around 102.4 ± 15.2 nm and a homogeneous dispersion with 1–2 nm thickness, showing single- or double-layered graphene structures. Glutathione (GSH) or dithiothreitol (DTT) was used as a redox-responsive cleavable disulfide linker. These compounds did not present any fluorescence and phototoxic properties, even upon light irradiation. The UV/Vis and fluorescence spectroscopy studies demonstrated the quenching effect of GO on the fluorescence yield of Ce6 and the cleavage of the disulfide bonds. The fluorescence intensity of GO–SS–Ce6 was ninefold greater after incubation in 1 mM DTT solution for 3 h. The authors also studied the ^1^O_2_ generation of several solutions (free Ce6, GO–SS–Ce6, and GO–SS–Ce6 + 1 mM DTT) under CW laser beam irradiation (670 nm, 100 mW/cm^2^) and found that the simultaneous treatment of GO–SS–Ce6 by 1 mM DTT and laser irradiation produces a significant increase of ^1^O_2_ generation compared to the GO–SS–Ce6 solution alone, reaching around the same level as the free Ce6 solution. The in vitro GSH-activatable fluorescence imaging and PDT studies of free Ce6 and GO–SS–Ce6 towards human lung cancer A549 cells (dark incubation for 24 h followed by diode laser irradiation at 670 nm, 20 J/cm^2^) showed effective cellular internalization and preferential accumulation of Ce6 in the cancer cells, especially in the lysosomes (Fig. 42).

Tian et al. (2015) synthesized GO coupled to FA, a PS, and to an enzyme-responsive substrate (peptide) to control the selective release of PS at specific sites. The folate-based conjugate and the PS-labeled peptide substrate consist of 1,2-distearoyl-sn-glycero-3-phosphoethanolamine-*N*-[folate(poly ethylene glycol)-2000] (DSPE-PEG2000-FA) and Ce6-GRRGKGGFFFF (Ce6-Pep), respectively, and were loaded onto GO by *π*–*π* and hydrophobic interactions. The peptide GRRGKGGFFFF is known to be a cathepsin B (CaB)-activatable substrate with a specific cleavage of the RR peptide bond. GO sheets have a size less than 100 nm and a dispersion with 0.4 nm thickness, showing a single-layered graphene structure. The successive loading of Ce6-Pep and DSPE-PEG2000-FA was observed with the increase of thickness, rising from 0.4 to 0.7 then to 1.2 nm, respectively. The Ce6-Pep loading of Ce6-Pep/DSPE-PEG2000-FA/GO nanoprobe was 35 wt%. The fluorescence intensity of Ce6-Pep/GO increased by a factor of 18 after the addition of CaB, the fluorescence of Ce6-Pep was not restored by adding Cathepsins D or L with Ce6-Pep/GO. Lysosome-targeting and CaB-activatable fluorescence nanoprobes for imaging cancer cells have been shown with in vitro studies using FR-positive HeLa or KB cells and FR-negative HaCaT or A549 cells. Other in vitro and in vivo studies (HaCaT and/or HeLa cells) were also performed to demonstrate the PDT effect (660 nm, 250 mW/cm^2^, 50 J/cm^2^ during 200 s) and in vivo tumor imaging.

#### PDT and PTT applications

**Fig. 43:**
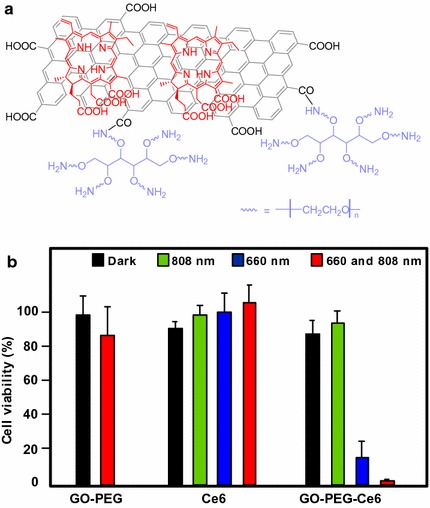
**a** Schematic structure of GO-PEG–Ce6. **b** Viability of KB cells under different light irradiations (Ce6 concentration: 5 μM) [Taken from reference (Tian et al. 2011)]

Tian and co-workers (2011) combined the PTT effect and PDT using PEGylated graphene oxide (GO-PEG) loaded with Ce6 via supramolecular *π*–*π* stacking. The size of the NPs was down to 50 nm and the thickness was about 1 nm. The maximal Ce6 loading capacity of GO-PEG was 15 wt% at the feeding concentration of 3 mM. The in vitro study was carried out after the exposure of KB cells to GO-PEG–Ce6 followed by different possible laser irradiations: (i) at 660 nm (15 J/cm^2^) for 5 min to test the PDT effect, (ii) at 808 nm (0.3 W/cm^2^, 360 J/cm^2^, 20 min) to test the PTT effect, or (iii) both with PTT irradiation before PDT treatment. Despite a reduction of the ^1^O_2_ generation for GO-PEG–Ce6 compared to free Ce6, the photodynamic effect was significantly increased thanks to a synergistic combination of PDT and PTT effects (Fig. 43).

Cao and co-workers (2016) used the same GO-PEG–Ce6 NPs to apply tandem PDT/PTT treatment on the murine breast cancer cell line 4T1. The authors obtained similar results to Tian et al. (vide supra). Interestingly, after PTT treatment they observed a greater tumor apparent diffusion coefficient (ADC) value in diffusion-weighted imaging (DWI) maps. Furthermore, after PDT treatment the apparent transverse relaxation rate (*R*_2_*) was enhanced and allowed blood oxygenation level-dependent magnetic resonance imaging (BOLD MRI). They clearly demonstrated that the tandem DWI and BOLD MRI after PTT/PDT treatment is a very promising tool for monitoring and prognosis.

**Fig. 44:**
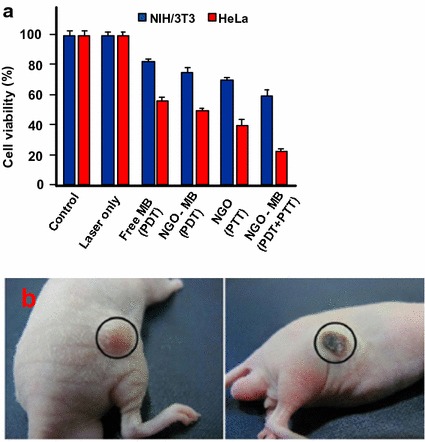
**a** In vitro cytotoxicity effect of MB, NGO, and NGO–MB in NIH/3T3 and HeLa cells after PDT, PTT, and PDT + PTT treatments. **b** PTT treatment effect on the tumor tissue before (left) and after 1-day irradiation (right) [Reused with permission from reference (Sahu et al. 2013)]

Sahu et al. (2013) developed nano-graphene oxide (NGO) coated with pluronic block copolymer to stabilize the NPs in biological fluids. The hydrodynamic size of NGO was 38.4 ± 3.1 nm, while it was 40.6 ± 2.8 nm after coating with pluronic. Methylene blue (MB) was loaded onto the coated NGO (NGO–MB) via electrostatic interactions. The maximal MB loading capacity was 22.7 ± 0.6 wt% at the feeding ratio of 70:30 (NGO/MB) with a hydrodynamic size of 43.5 ± 5.1 nm and large aggregates were observed when the feeding ratio is 50:50. The in vitro study (PDT irradiation: CW laser at 655 nm for 3 min, PTT irradiation: NIR laser at 808 nm for 3 min or dual treatment) was performed with human cervical cancer HeLa cells and mouse normal fibroblast cells (NIH/3T3) and the in vivo study (PDT irradiation: CW laser at 650 nm, ≈ 150 mW/cm^2^, for 10 min; PTT irradiation: NIR laser at 808 nm, 2 W/cm^2^ for 3 min) with HeLa cells. NGO–MB generates less ^1^O_2_ than free MB but, as seen previously, the photodynamic effect is dramatically increased in vitro with dual treatment (Fig. 44a). Concerning the in vivo photodynamic effect on mice, the PDT treatment showed a small decrease in tumor growth, while it has been significantly reduced by the PTT treatment (incomplete tumor ablation) (Fig. 44b). The combination of PDT/PTT treatments revealed a complete regression of tumor even after 15 days of treatment.

**Fig. 45:**
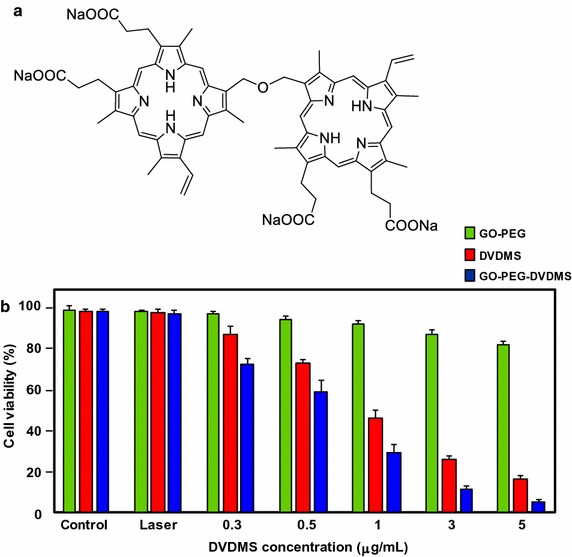
**a** Chemical structure of DVDMS. **b** Viability of U87MG cells incubated with various concentrations of free DVDMS, GO-PEG, or GO-PEG-DVDMS after irradiation by 630-nm laser (2 J) [Taken from references (Yan et al. 2015a, b)]

In two recent publications (Yan et al. 2015a, b), Chen’s team described new nanocomposites made from a PEGylated graphene oxide (PEG–GO) loaded by sinoporphyrin sodium (DVDMS, Fig. 45a). The DVDMS loading capacity reaches a maximum of 201.2 wt% in the resulting GO-PEG–DVDMS nanodevice. Interestingly, the fluorescence intensities of GO-PEG–DVDMS are 3–8 times higher than that of the free DVDMS. This can be due to the two sterically hindered porphyrin rings in the PS, one being involved in the *π*–*π* interactions with GO, the other being hanging out and responsible for this phenomenon. ^1^O_2_ generation of GO-PEG–DVDMS is slightly lower than that of DVDMS at the same concentration. In the first paper (Yan et al. 2015a), the authors showed a slight dose-dependent in vitro cytotoxicity to U87MG cells in the range of 0.3–5 mg/mL of DVDMS (Fig. 45b). Concerning the PDT efficiency, they found that GO-PEG–DVDMS induced quasi-total cell death at the dose of 5 mg/mL of DVDMS upon 630-nm laser irradiation, after 24-h incubation. The in vivo PDT experiments were performed in U87MG tumor-bearing mice model (630 nm at a dose of 50 J) and the GO-PEG–DVDMS laser group showed a life span longer than 30 days as compared to 22 days for the DVDMS laser group.

**Fig. 46:**
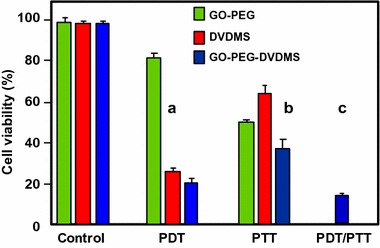
Effect of irradiation conditions (PDT and/or PTT) on HeLa cell viability using free DVDMS, GO-PEG, or GO-PEG–DVDMS (m_GO-PEG_:m_DVDMS_ = 1:2, GO-PEG concentration = 3 μg/mL). a PDT effect (630 nm, 2 J/well). b PTT effect (808 nm, 1 W/cm^2^, 3 min/well). c Synergetic PDT/PTT effect [Taken from reference (Yan et al. 2015b)]

In the second paper (Yan et al. 2015b), they studied the dual-modality imaging-guided synergistic PDT/PTT therapies on PC9 cells in vitro and PC9 tumor-bearing mice in vivo. They noticed a high accumulation of GO-PEG–DVDMS in the tumor tissues due to EPR effect. 24 h post injection, PTT alone (808 nm, 1 W/cm^2^ for 10 min) induced an increase of tumor temperature up to 57 °C, while that of the healthy tissues only reached 32 °C. The synergistic effect of PDT/PTT is shown in Fig. 46.

**Fig. 47:**
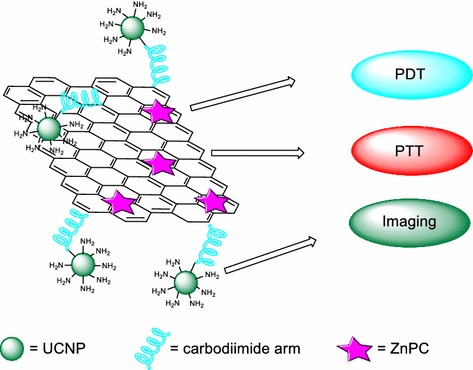
Schematic illustration of UCNPs–NGO/ZnPc as a multifunctional theranostic nanoplatform for cancer treatment [Taken from reference (Wang et al. 2013)]

Wang et al. (2013) developed a new concept of NIR imaging and PTT/PDT therapy. Upconversion nanoparticles (UCNPs) were obtained as previously described (Bogdan et al. 2011) and treated by poly(allylamine) as a surface coating agent. The modified UCNPs were then covalently coupled to NGO via carbodiimide cross-linking. The UCNPs–NGO was mixed with ZnPc to give the final composition (Fig. 47) with a ZnPc loading amount of 11 wt% and an average diameter of 40 nm as assessed by TEM.

**Fig. 48:**
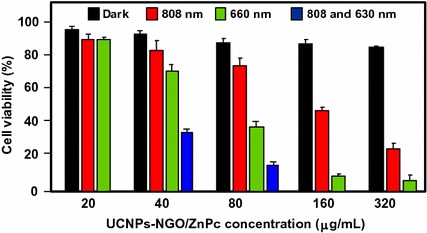
In vitro cell viability assay in HeLa cells of UCNPs–NGO/ZnPc at different concentrations without irradiation (black) and after PDT (green), PTT (red), or combined PDT/PTT (blue) treatments [Taken from reference (Wang et al. 2013)]

The ^1^O_2_ production (DPBF (1,3-diphenylisobenzofuran) method) of UCNPs–NGO/ZnPc was lower than that of ZnPc alone due to the presence of NGO but was still 60%. Furthermore, the good biocompatibility and the low cytotoxicity allowed using these NPs for PDT treatment. Figure 48 shows the PDT and PTT effects after irradiation. It can be seen clearly that the PDT effect (green) is the main phenomenon as compared to the PTT effect (red), but the combined PDT/PTT effect reduced the viability of HeLa cells to 15% (blue).

**Fig. 49:**
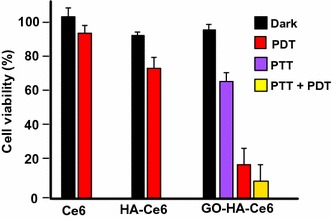
In vitro cell viability assay in A549 cells of free Ce6, HA–Ce6, and GO–HA–Ce6 at the same Ce6 concentration (1.8 μM equivalent) without irradiation (black) and after PDT (670 nm, red), PTT (810 nm, purple), or combined PDT/PTT (670 and 810 nm, yellow) treatments [Taken from reference (Cho et al. 2013)]

One year later, the same team (Cho et al. 2013) described the elaboration of GO–HA–Ce6 nanoplatform, as was the case with Li et al. (2013), but this time based on a covalently linked hyaluronic acid–chlorin e6 (HA–Ce6) loaded onto the surface of nanosized GO via *π*–*π* and hydrophobic interactions (average hydrodynamic size 441.1 ± 48.8 nm). The objective of this work was to develop an enzyme-activatable theranostic agent using HA, which is known to preferentially accumulate in the extracellular matrix and to be degraded quickly in the presence of hyaluronidase (HAdase) enzyme. The quenching effect of GO on the fluorescence yield of Ce6 and the cleavage of the glycosidic bonds were demonstrated by comparing the fluorescence emission spectra of GO, HA–Ce6, and GO–HA–Ce6 at a same Ce6 concentration. When GO–HA–Ce6 is treated with HAdase (800 U/mL), the fluorescence signal increased by a factor of 5, indicating a Ce6 release of approximately 21% from the GO surface. The in vitro studies (Fig. 49) of GO–HA–Ce6 were performed with human lung cancer A549 cells and a synergistic phototherapy effect (PDT: CW laser beam irradiation, 670 nm, 50 mW/cm^2^, 4 J/cm^2^ and PTT: 810 nm, 4 W/cm^2^, 250 J/cm^2^) was observed, making it an interesting HAdase-activable theranostic agent combining phototherapy (PDT/PTT) and activatable fluorescence imaging-guided treatment for cancer tumors.

**Fig. 50:**
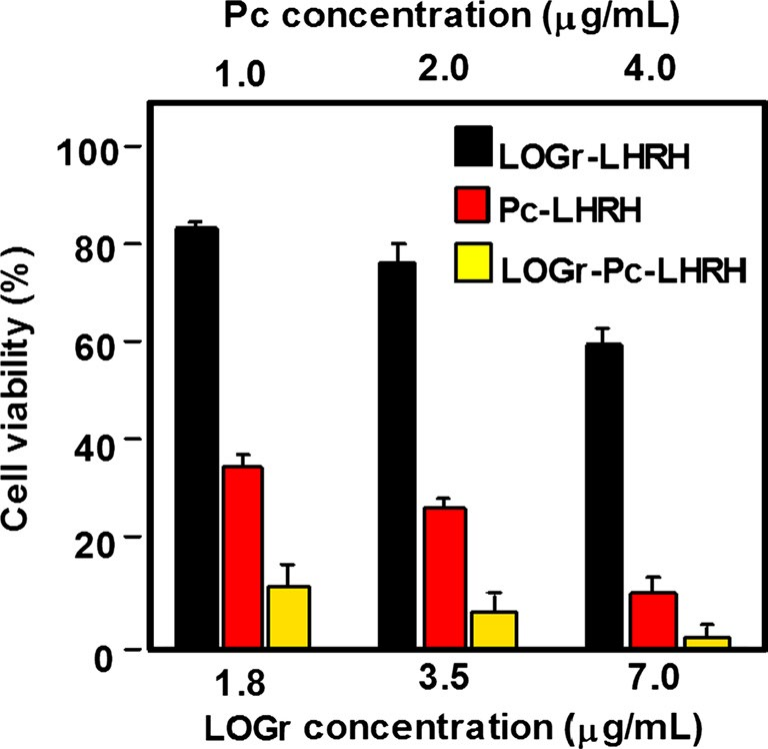
In vitro cell viability assay in A2780/AD cells of LOGr–LHRH, Pc–LHRH, and LOGr–Pc–LHRH at different LOGr concentrations after combined PDT/PTT treatment (690 nm, 0.3 W/cm^2^, 15 min) [Taken from reference (Taratula et al. 2015)]

Taratula and co-workers (2015) described a new targeted graphene-based nanoplatform combining phototherapy (PDT/PTT) and imaging-guided tumor treatment for ovarian cancer. For the first time, the monosubstituted silicon phthalocyanine Pc(OH)(mob) was loaded onto polypropylenimine generation 4 (PPIG4) dendrimer and the resulting adduct was covalently attached to low-oxygen graphene (LOGr) nanosheets through the amine functions of the dendrimer affording LOGr–Pc. Finally, the luteinizing hormone-releasing hormone (LHRH) peptide was grafted to give LOGr–Pc–LHRH nanoplatform. PEGylation and peptide were necessary to increase biocompatibility and to target LHRH receptors overexpressed on the membranes of both primary and metastatic ovarian cancer cells. The hydrodynamic diameter of LOGr–PC–LHRH was 78.3 ± 9.54 nm (≈ 15 and 62 nm for the LOGr and Pc–LHRH, respectively) and the Pc loading efficiency onto PPIG4 was approximately 20 wt%. The in vitro combination phototherapy effect (PDT/PTT) of the nanoplatform ([Pc] = 1.0–4.0 μg/mL and [LOGr] = 1.8–7.0 μg/mL) towards LHRH-positive A2780/AD ovarian cancer cells has been demonstrated using a single wavelength (laser diode irradiation at 690 nm, 0.3 W/cm^2^ for 15 min) to induce ^1^O_2_ generation by Pc and PTT effect by LOGr (Fig. 50). The in vivo NIR fluorescence imaging was also highlighted after administration of LOGr–Pc–LHRH in mice bearing A2780/AD tumor.

**Fig. 51:**
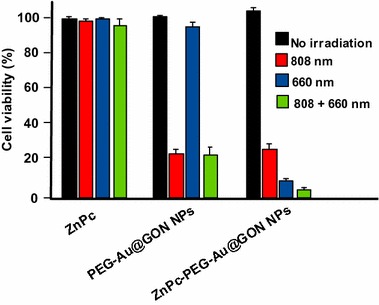
In vitro cell viability assay in HeLa cells of free ZnPc, PEG–Au@GON NPs, and ZnPc–PEG–Au@GON NPs without irradiation (black) and after PDT (660 nm, 0.2 W/cm^2^, 10 min, blue), PTT (808 nm, 0.67 W/cm^2^, 20 min, red), or combined PDT/PTT (green) treatments [Taken from reference (Kim et al. 2015)]

In the same year, Kim and co-workers (2015) opted to use core and shell NPs (core@shell) to report a “one-pot synthesis” of a new theranostic nanoplatform combining Raman bioimaging and phototherapy (PDT/PTT). These core@shell NPs are composed of Au core and graphene oxide nanocolloid (GON) shells, onto which PEG and ZnPc have been grafted and loaded, respectively. The PEG was added to give a better colloidal stability in biological media and the PS was loaded onto Au@GON NPs via *π*–*π* interactions (loading efficiency 1.9 × 10^6^ ZnPc/NP). The size of the Au@GON NPs, approximately spherical in shape, is 60 ± 15 nm with 2–3 nm GON shell thickness. The in vitro evaluation of Raman imaging and dual PDT/PTT effect of the resulting ZnPc–PEG–Au@GON NPs in human cervical cancer HeLa cells were investigated and the results demonstrated that these NPs can be intended to conduct Raman imaging and have a synergistic phototherapy effect (PDT: LED irradiation at 660 nm, 0.2 mW/cm^2^ for 10 min and PTT: laser irradiation at 808 nm, 0.67 W/cm^2^ for 20 min) with ^1^O_2_ generation (Fig. 51).

Graphene is a new material but is already called a new wonderful material thanks to many advantages. Its structure and physico-chemical properties make it an excellent candidate for different applications ranging from solar cells to cancer treatment. In the field of PDT, the first paper was published in 2010 and more than 35 papers have been published to date.

Thanks to its large surface area, it is possible to use it as an efficient PS carrier. Its *π*–*π* structure can easily bind to aromatic PS. Graphene presents also high dispersibility in water and good stability in biological media. We can notice that it is a very good conductor of heat and it can be used to perform both PDT and PTT. One of the disadvantages might be its size compared to small NPs, except nanorods.

One of the advantages of graphene oxide (GO) is its easy dispersibility in water and other organic solvents due to the presence of oxygen atoms. Moreover, it can be very easily functionalized and also one publication describes the covalent binding of Ce6 onto GO. Folic acid, hyaluronic acid, or peptides have already been successfully bound onto GO.

Few papers describe the use of reduced graphene oxide (rGO), which might be due to its tendency to aggregate. Table 7 below summarized the data available on the application of graphene NPs with grafted or encapsulated PSs in PDT and/or PTT.

**Table 7:** Application of graphene NPs in PDT and/or PTT

Type of NPs (size, nm)	PS (amount)	NPs–PS interactions	Irradiation conditions	Type of ROS	Cancer cell line		Ref.
					In vitro	In vivo	
Nanosheets (200)	ZnPc (14 wt%)	*π*–*π* stacking Hydrophobic	UV band-path filtered Xe light, 60 J/cm^2^, 10 min	nd	MCF-7	‒	Dong et al. (2010)
Nanosheets (nd)	MB (nd)	nd	Laser 785 nm	^1^O_2_	‒	‒	Wojtoniszak et al. (2013)
Nanosheets (148)	Ce6 (51.9 wt%)	*π*–*π* stacking Hydrophobic	LED 660 nm, 8000 mCd, 30 min	nd	SCC7	SCC7 bearing mice C3H/HeN mice	Miao et al. (2013)
Nanosheets (20–40)	Ce6 (26 wt%)	*π*–*π* stacking Hydrophobic	Laser 660 nm, 0.2 W/cm^2^, 5 min	ROS ^1^O_2_	HeLa	‒	Zeng et al. (2015)
Nanosheets (nd)	Ce6 (160 wt%)	*π*–*π* stacking	Laser 660 nm, 2 min	ROS	HeLa	‒	Liu et al. (2015a)
Nanosheets (200)	ZnPc (60 wt%)	*π*–*π* stacking Hydrophobic	Light 660 nm, 0.15 W/cm^2^, 10 min	^1^O_2_	HeLa MCF-7	‒	Wu et al. (2014)
Nanosheets (120)	MitoTPP (37.2 wt%)	*π*–π stacking Electrostatic	LED 650 nm, 10 mW/cm^2^, 30 min	^1^O_2_	HeLa	‒	Xu et al. (2015)
Nanosheets (nd)	HP (23.6 wt%)	*π*–*π* stacking Hydrophobic	Laser 671 nm, 0.1 W/cm^2^, 5 min	^1^O_2_	HeLa	‒	Zhou et al. (2015)
Nanosheets (300–400)	AdaTPP (nd)	*π*–*π* stacking	Visible light	ROS	HeLa OCT-1	HeLa-bearing BALB/c nude mice	Yang et al. (2012)
Nanosheets (80)	AP-Ce6 (23.1 wt%)	*π*–*π* stacking	Light 650 nm, 50 mW/cm^2^, 20 min	^1^O_2_	HepG2	‒	Yan et al. (2014)
Nanosheets (nd)	Ce6 (80 wt%)	*π*–*π* stacking Hydrophobic	He–Ne laser 632.8 nm, 30 mW/cm^2^, 10 min	nd	MGC803	‒	Huang et al. (2011)
Nanosheets (nd)	Ce6 (80 wt%)	*π*–*π* stacking Hydrophobic	Laser 671 nm, 30 mW/cm^2^, 3 min	^1^O_2_	MGC803	‒	Huang et al. (2015)
Nanosheets (78.1)	Ce6 (115 wt%)	*π*–*π* stacking Hydrophobic	Laser 670 nm, 1.8 J/cm^2^	^1^O_2_	HeLa	‒	Li et al. (2013)
Nanosheets (50)	Ce6 (15 wt%)	*π*–*π* stacking	Laser 660 nm, 15 J/cm^2^, 5 min (PDT); Laser 808 nm, 0.3 W/cm^2^, 360 J/cm^2^ (PTT)	^1^O_2_	KB	‒	Tian et al. (2011)
Nanosheets (38.4)	MB (22.7 wt%)	Electrostatic	CW laser 650 nm, 0.15 W/cm^2^, 10 min (PDT) ; NIR laser 808 nm, 2 W/cm^2^, 3 min (PTT)	^1^O_2_	HeLa NIH/3T3	HeLa	(Sahu et al. 2013)
Nanosheets (nd)	TSCuPc (27 wt%)	*π*–*π* stacking	Laser 650 nm, 3 W/cm^2^, 5 min	ROS	HeLa	‒	Jiang et al. (2014b)
Nanorods (nd)	TSCuPc (38 wt%)	*π*–*π* stacking	Laser 650 nm, 3 W/cm^2^, 5 min	ROS	HeLa	‒	Jiang et al. (2014a)
Nanosheets (20.5)	DVDMS (100 wt%)	*π*–*π* stacking	Laser 630 nm, 50 J (PDT); NIR laser 808 nm, 1 W/cm^2^, 10 min (PTT)	^1^O_2_	PC9	PC9 tumor-bearing mice	Yan et al. (2015a)
Nanosheets (50)	DVDMS (201.2 wt%)	*π*–*π* stacking Hydrophobic	Laser 630 nm, 50 J	^1^O_2_	U87MG	U87MG tumor-bearing mice	Yan et al. (2015b)
Nanorods (1–5)	ZnMAPc (nd)	nd	Diode laser 676 nm, 98 mW/cm^2^, 5 J/cm^2^	^1^O_2_	A375	‒	Ogbodu et al. (2013a, b, 2015b)
Nanorods (1–5)	ZnMAPc (nd)	nd	602 nm	^1^O_2_	‒	‒	Ogbodu et al. (2013b)
Nanorods (1–5)	ZnMCPPc (nd)	nd	Quartz lamp 600–1000 nm, 28–112 J/cm^2^, 5–20 min	^1^O_2_	MCF-7	‒	Ogbodu et al. (2015a)
Nanorods (1–5)	ZnOCPc (nd)	nd	610 nm	^1^O_2_	‒	‒	Ogbodu and Nyokong (2014)
Nanorods (nd)	POR, MYR (nd)	*π*–*π* stacking	LED lamp 570 nm (yellow filter), 630 nm (red filter) or 940 nm	^1^O_2_	‒	‒	Safar et al. (2015)
Nanorods (nd)	Ce6 (11.3 wt%)	*π*–*π* stacking	Laser 630 nm, 0.15 W/cm^2^, 10 min	nd	HeLa NIH/3T3	‒	Xiao et al. (2012)
Nanosheets (40)	ZnPc (11 wt%)	*π*–*π* stacking	Laser 630 nm, 50 mW/cm^2^, 10 min (PDT); NIR laser 808 nm, 2 W/cm^2^, 10 min (PTT)	^1^O_2_	HeLa KB	‒	Wang et al. (2013)
Hydrogel shells (35)	AE (nd)	*π*–*π* stacking Hydrophobic	Laser 600 nm, 0.2 W/cm^2^, 10 min	^1^O_2_	HeLa CHO	‒	Chang et al. (2015)
nd (40)	SiNc4 (8.5 wt%)	*π*–*π* stacking	Halogen lamp 775 nm, 0.3 W/cm^2^, 60 min	ROS ^1^O_2_	HeLa	‒	Gollavelli and Ling (2014)
Nanosheets (50)	HPPH (131 wt%)	*π*–*π* stacking	Laser 671 nm, 2-8 mW/cm^2^, 3 min (in vitro) and 75 mW/cm^2^, 20 min (in vivo)	^1^O_2_	4T1	4T1-bearing mice	Rong et al. (2014)
Nanosheets (102.4)	Ce6 (nd)	Grafted with disulfide bond (SS)	CW laser beam 670 nm, 0.1 W/cm^2^, 20 J/cm^2^	^1^O_2_	A549	‒	Cho and Choi (2012)
Nanosheets (441.1)	Ce6 (nd)	Hydrophobic	CW laser beam 670 nm, 0.1 W/cm^2^, 20 J/cm^2^	^1^O_2_	A549	‒	Cho et al. (2013)
Nanosheets (100)	Ce6-Pep (35 wt%)	*π*–*π* stacking Hydrophobic	Laser 660 nm, 0.25 W/cm^2^, 200 s	^1^O_2_	KB A549 HeLa HaCaT	HeLa-bearing mice	Tian et al. (2015)
Nanosheets (15)	PcSi(OH)(mob) (20 wt%)	Encapsulated into PPIG4 dendrimer	Laser diode 690 nm, 0.3 W/cm^2^, 15 min (PDT/PTT, in vitro)	^1^O_2_	A2780/AD	A2780/AD-bearing mice	Taratula et al. (2013, 2015)
Nanocolloid Core@Shell NPs (15)	ZnPc (1.9 10^6^ ZnPc/Au@GON NP)	*π*–*π* stacking	LED 660 nm, 0.2 W/cm^2^, 10 min (PDT); NIR laser 808 nm, 0.67 W/cm^2^, 20 min (PTT)	^1^O_2_	HeLa	‒	Kim et al. (2015)

*NPs* nanoparticles, *PS* photosensitizer, *ROS* reactive oxygen species, *nd* not disclosed, *ZnPc* zinc phthalocyanine, *UV* ultraviolet, *MB* methylene blue, *Ce6* chlorin e6, *MitoTPP* 5-(*p*-(4-bromo)butoxyphenyl)-10,15,20-triphenylporphyrin, *HP* hematoporphyrin, *AdaTPP* adamantanyl porphyrin, *AP* aptamer, *CW* continuous wave, *TSCuPc* tetrasulfonic acid tetrasodium salt copper phthalocyanine, *DVDMS* sinoporphyrin sodium, *NIR* near-infrared, *ZnMAPc* zinc monoamino phthalocyanine, *ZnMCPPc* zinc monocarboxyphenoxy phthalocyanine, *ZnOCPc* zinc octacarboxy phthalocyanine, *POR meso*-tetrakis(4-pyridyl)porphyrin tosylate salt, *MYR meso*-tetrakis(*N*-myristyl-4-pyridinium)porphyrin tosylate salt, *AE* chlorophyll derivatives in amaranth extract, *SiNc4* silicon napthalocyanine bis (trihexylsilyloxide), *HPPH* 2-(1-hexyloxyethyl)-2-devinyl pyropheophorbide-alpha, *SS* disulfide, *HA* hyaluronic acid, *Pep* peptide, *PcSi(OH)(mob)* monosubstituted phthalocyanine derivative, *PPIG4* polypropylenimine generation 4, *GON* graphene oxide nanocolloid

## Conclusions

Nanoparticles (NPs) hold great promise for the design of new compounds for PDT. In this field, many biodegradable NPs have been formulated with the problem of controlling the NPs’ degradation and the release of the PS. Non-degradable NPs such as TiO_2_, ZnO, fullerene, and graphene are very promising NPs for PDT applications even if they are already well known in the field of photocatalysis. Their small size, low toxicity, and easy functionalization make them very good candidates for bioimaging and cancer therapy (PDT and/or PTT treatments). According to the results presented in this review and in the best of worlds, the ideal approach would allow us to develop theranostic platforms combining both bioimaging to localize tumor cells and a dual PDT/PTT treatment to eradicate tumor cells using a single wavelength while minimizing as much as possible the side effects. In order to achieve this ambitious goal, it will be necessary to modify the NP surface with (1) a contrast agent suitable for MRI, PET, or X-ray imaging, (2) PDT and PTT agents having photodynamic and photothermal activity in hypoxic and non-hypoxic conditions, and (3) a vector to target tumor cells.

## Abbreviations

ABDA: 9,10-anthracenediyl-bis(methylene)dimalonic acid
AcTMP: 5-(4-acetamidophenyl)-10,15,20-tris(4-methoxyphenyl)porphyrin
AdaTPP: adamantanyl porphyrin
ADC: apparent diffusion coefficient
AE: amaranth extract
AFM: atomic force microscopy
5-ALA: 5-aminolevulinic acid
AlPcS_4_: aluminum phthalocyanine chloride tetrasulfonate
AP: aptamer
APTES: 3-aminopropyltriethoxysilane
ATMP: 5-(4-amidophenyl)-10,15,20-tris(4-methoxyphenyl)porphyrin
ATP: adenosine-5′-triphosphate
βCD: beta-cyclodextrin
BDP: boron-dipyrromethene
BOLD: blood oxygenation level-dependent
BPEI: branched polyethylenimine
CaB: cathepsin B
CBC: complete blood cell count
CC_50_: cytotoxic concentration that induces 50% of cell death
Ce6: chlorin e6
CM-H_2_DCFDA: 2,7-dichlorodihydrofluorescein diacetate acetyl ester
CW: continuous wave
DBMC-Cmbl: 7,8-dihydroxy-4-bromomethylcoumarin-chlorambucil
DCFH-DA: 2′,7′-dichlorodihydrofluorescein diacetate
DLS: dynamic light scattering
DMF: dimethylformamide
DNA: deoxyribonucleic acid
Dox: doxorubicin
DPBF: 1,3-diphenylisobenzofuran
DSPE: 1,2-distearoyl-sn-glycero-3-phosphoethanolamine
D-TMPyP: 5-(4-formylphenyl)-10,15,20-tris(4-pyridyl)-porphyrin
DTT: dithiothreitol
DTX: docetaxel
DVDMS: sinoporphyrin sodium
DWI: diffusion-weighted imaging
EPR: enhanced permeability and retention
E-SWCNT: chirality-enriched (6,5) single-walled carbon nanotube
FA: folic acid
FDA: Food and Drug Administration
FCNV: fullerene C_70_ nanovesicle
FP: fine particle
FRET: fluorescence resonance energy transfer
FTEP: 5-(4-formylphenyl)-10,15,20-tris[3-(*N*-ethylcarbozoyl)] porphyrin
FTIR: Fourier transform infrared
G: graphene
GO: graphene oxide
GON: graphene oxide nanocolloid
GR: graphene sheets
GSH: glutathione
HA: hyaluronic acid
HP: hematoporphyrin
HPPH: 2-(1-hexyloxyethyl)-2-devinyl pyropheophorbide-alpha
H_2_TM4PyP (OTs)_4_: *meso*-tetrakis(4-pyridyl)porphyrin tosylate salt (POR)
H_2_TMy4PyP (OTs)_4_: *meso*-tetrakis(*N*-myristyl-4-pyridinium)porphyrin tosylate salt (MYR)
IC_50_: concentration that induces 50% of parasite inhibition
IR: infrared
LED: light-emitting diode
LHRH: luteinizing hormone-releasing hormone
LOGr: low-oxygen graphene
MA: malonic acid derivatives
MB: methylene blue
MFG: magnetic and fluorescent graphene
MitoTPP: 5-(p-(4-trimethylammonium)-butoxyphenyl)-10,15,20-triphenylporphyrin bromide
mPEG: methoxy-poly(ethylene glycol)
MRI: magnetic resonance imaging
MTAP: *meso*-tetra(*o*-aminophenyl)porphyrin
MTCP: *meso*-tetra(4-carboxyphenyl)porphyrin
MTT: methylthiazolyl tetrazolium
NBT: nitroblue tetrazolium
NGO: nano-graphene oxide
NHS: *N*-hydroxysuccinimide
NIR: near-infrared
NP: nanoparticle
NR: nanorod
N-TiO_2_: nitrogen-doped TiO_2_
NV: nanovesicle
NW: nanowhisker
(OH)AlPcSmix: mixture of the di-, tri-, and tetra-sulfonated phthalocyanine derivatives
PBS: phosphate buffer saline
Pc: phthalocyanine
Pc(OH)(mob): monosubstituted silicon phthalocyanine
P–C_60_: porphyrin–[C_60_] fullerene dyads
P–C_70_: porphyrin–[C_70_] fullerene dyads
PEG: polyethylene glycol
pGO: polyethylene glycol-grafted graphene oxide
PLA: polylactide
PLL: polylysine
PS: photosensitizer
PDT: photodynamic therapy
Pep: peptide
PET: positron emission tomography
*Φ*_Δ_: singlet oxygen production quantum yield
PPDME: protoporphyrin dimethyl ester
PPIG4: polypropylenimine generation 4
PpIX: protoporphyrin IX
PTT: photothermal therapy
PVP: poly(vinylpyrrolidone)
Pyro: pyropheophorbide
rGO: reduced graphene oxide
ROS: reactive oxygen species
SEM: scanning electron microscopy
Sil: silane arm
SiNc_4_: silicon napthalocyanine bis(trihexylsilyloxide)
SLPDT: self-lighting photodynamic therapy
SOG: singlet oxygen generation
SOSG: singlet oxygen sensor green
SWNH: single-walled carbon nanohorn
SWCNT: single-walled carbon nanotube
TEM: transmission electron microscopy
TFC_70_: tri-malonate derivative of fullerene C_70_
TMPyP: trismethylpyridylporphyrin
TPP: tetraphenylporphyrin
TSCuPc: tetrasulfonic acid tetrasodium salt coper phthalocyanine
TSPP: *meso*-tetra(4-sulfonatophenyl)porphyrin
TTA-UC: triplet–triplet annihilation upconversion
UCNP: upconversion nanoparticles
UV: ultraviolet
VER: verteporfin
Vis: visible
WST-1: water-soluble tetrazolium salt
wt: weight
ZnMAPc: zinc monoamino phthalocyanine
ZnMCPPc: zinc monocarboxyphenoxy phthalocyanine
ZnOPc: zinc octacarboxy phthalocyanine
ZnPc: zinc phthalocyanine
ZnTMAAPc: 2,(3),9(10),16(17),23(24)-tetrakis-(mercaptoacetic acid phthalocyaninato) zinc(II)
ZnTMPAPc: 2,(3),9(10),16(17),23(24)-tetrakis-(mercaptopropanoic acid phthalocyaninato) zinc(II)
